# Molecular and cellular characterization of two patient-derived ductal carcinoma in situ (DCIS) cell lines, ETCC-006 and ETCC-010

**DOI:** 10.1186/s12885-021-08511-2

**Published:** 2021-07-08

**Authors:** Julia Samson, Magdalina Derlipanska, Oza Zaheed, Kellie Dean

**Affiliations:** 1 School of Biochemistry and Cell Biology, Western Gateway Building, University College Cork, Cork, T12XF62 Ireland; 2 Present address: EFOR, 25-29 Rue Anatole France, 92300 Levallois-Perret, France

**Keywords:** Ductal carcinoma in situ (DCIS), Breast carcinoma cell lines, qRT-PCR, RNA sequencing (RNAseq), Proliferation, Migration, Anchorage-independent growth, Epithelial to mesenchymal transition, Cell signalling pathways, Cell cycle

## Abstract

**Background:**

Currently it is unclear how in situ breast cancer progresses to invasive disease; therefore, a better understanding of the events that occur during the transition to invasive carcinoma is warranted. Here we have conducted a detailed molecular and cellular characterization of two, patient-derived, ductal carcinoma in situ (DCIS) cell lines, ETCC-006 and ETCC-010.

**Methods:**

Human DCIS cell lines, ETCC-006 and ETCC-010, were compared against a panel of cell lines including the immortalized, breast epithelial cell line, MCF10A, breast cancer cell lines, MCF7 and MDA-MB-231, and another DCIS line, MCF10DCIS.com. Cell morphology, hormone and HER2/ERBB2 receptor status, cell proliferation, survival, migration, anchorage-independent growth, indicators of EMT, cell signalling pathways and cell cycle proteins were examined using immunostaining, immunoblots, and quantitative, reverse transcriptase PCR (qRT-PCR), along with clonogenic, wound-closure and soft agar assays. RNA sequencing (RNAseq) was used to provide a transcriptomic profile.

**Results:**

ETCC-006 and ETCC-010 cells displayed notable differences to another DCIS cell line, MCF10DCIS.com, in terms of morphology, steroid-receptor/HER status and markers of EMT. The ETCC cell lines lack ER/PR and HER, form colonies in clonogenic assays, have migratory capacity and are capable of anchorage-independent growth. Despite being isogenic, less than 30% of differentially expressed transcripts overlapped between the two lines, with enrichment in pathways involving receptor tyrosine kinases and DNA replication/cell cycle programs and in gene sets responsible for extracellular matrix organisation and ion transport.

**Conclusions:**

For the first time, we provide a molecular and cellular characterization of two, patient-derived DCIS cell lines, ETCC-006 and ETCC-010, facilitating future investigations into the molecular basis of DCIS to invasive ductal carcinoma transition.

**Supplementary Information:**

The online version contains supplementary material available at 10.1186/s12885-021-08511-2.

## Background

Biomedical research relies heavily on the use of cellular in vitro models. In cancer research, a wide variety of cell lines are available to study the molecular details of cancer initiation and progression and are enormously beneficial to develop and test therapeutic drugs. For breast cancer research, MCF-7, T47D and MDA-MB-231 are utilized in more than two thirds of the studies using cell lines [1], with MCF-7 and T47D cell lines representing luminal type A breast cancer [2] and MDA-MB-231 representing basal-like type B [3]. MDA-MB-231 cells are used extensively as a model for triple-negative breast cancer, based on the lack of expression of cell surface receptors, estrogen receptor (ER), progesterone receptor (PR) and the growth factor receptor, human epidermal growth factor receptor-2 (HER2/ERBB2) [4]. While numerous cell lines have been developed to represent invasive ductal carcinoma (IDC), fewer lines are available for ductal carcinoma in situ (DCIS), a non-obligate precursor to invasive breast cancer [5]. DCIS results from an abnormal proliferation of epithelial cells in the duct of the breast and is associated with a lack of invasive growth into the local stroma [6, 7].

Some cellular models of early stage breast cancer have been developed. MCF10DCIS.com is the DCIS cell line most used by researchers according to the literature. MCF10DCIS.com was derived from a xenograft lesion following two trocar passages of the premalignant cell line MCF-10AT [8, 9]. In xenografts, MCF10DCIS.com forms lesions resembling comedo DCIS with central necrotic areas [9]. SUM225 is another available model for DCIS; it is derived from a chest wall recurrence in a patient diagnosed and treated for DCIS 6 years prior [10]. In 3D cultures, SUM225 forms atypical clusters [11] and using a mouse mammary intraductal-transplantation method, SUM225 formed lesions similar to human DCIS [12]. While those DCIS cell lines and a few others (reviewed by Brock et al. [13]) exist, none are directly derived from a primary DCIS tumor.

In 2014, Yong et al. [14] developed five DCIS sub-cell lines derived from a primary tumor by overexpressing the human telomerase reverse transcriptase (hTERT). For this study, we acquired two of those five cell lines, ETCC-006 and ETCC-010. We selected these two lines based on the original publication where the authors showed that each cell line had different abilities in proliferation and tumor growth when injected into mice, with ETCC-010 showing the most aggressive phenotype in vivo and ETCC-006 in vitro. Those cell lines have not been used in the literature other than in the original publication [13].

Yong et al. stated that the ETCC lines that they derived were ER positive, based on their observations using immunoblotting and immunofluorescence [14]. Leading on from their observations, we first aimed to stimulate those cells with estradiol and to analyze the changes in gene expression of estrogen responsive genes. However, in preliminary experiments we observed no difference in expression of estrogen responsive genes after estradiol stimulation; therefore, we considered whether our results might be explained by a lack of expression of ER in those cells. In consequence, we decided to conduct a molecular and cellular characterization of ETCC-006 and ETCC-010 DCIS cell lines to clarify and extend results published by Yong et al. [14] and to provide comprehensive information for our subsequent studies and to the research community. This work was conducted by comparing ETCC-006 and ETCC-010 to MCF10DCIS.com [9], the conventional cell line model of DCIS, and to the widely characterized breast cancer cell lines, MCF-7, a hormone-receptor positive breast cancer model [15, 16], MDA-MB-231, a triple-negative breast cancer model [4], and the immortalized, breast epithelial cell line MCF-10A [17].

In detail, we determined the cellular phenotypes of the two ETCC DCIS cell lines by examining general cell morphology, along with proliferation, survival, migration and anchorage-independent growth. Next we examined molecular features including, hormone-receptor status, markers for epithelial-mesenchymal transition (EMT) and protein expression levels of several signalling proteins in the AKT and ERK pathways and in the cell cycle. In parallel, RNA expression of several DCIS markers was measured by qRT-PCR and the transcriptomic profile of ETCC-006 and ETCC-010 was determined by RNA sequencing (RNAseq) and compared to an existing RNAseq data set from MCF-10A cells [17] and to RNAseq data sets from MCF10DCIS.com, MCF7 and MDA-MB-231 cells [18]. Overall, our results suggest that the ETCC-006 and ETCC-010 DCIS cell lines may represent a more aggressive form of DCIS and provide a valuable resource to examine the transition between non-invasive and invasive ductal carcinoma of the breast.

## Methods

### Cell culture

ETCC-006 and ETCC-010 were purchased from Leibniz-Institute DSMZ (Braunschweig, Germany). MCF10DCIS.com cells were obtained from Wayne State University (Michigan, USA). MCF-10A, MCF-7, MDA-MB-231, originally from ATCC, were a kind gift from Prof Rosemary O’Connor (UCC, Cork, Ireland). Cell lines were authenticated using short, tandem repeat (STR) profiling (Eurofins Genomics). Cells were cultured according to the following media requirements. MCF-10A cells were maintained in DMEM/F12 supplemented with 5% horse serum, 10 μg/ml insulin, 20 ng/ml EGF, 100 ng/ml cholera toxin and 0.5 μg/ml hydrocortisone. MCF-7 and MDA-MB-231 cells were cultured in DMEM supplemented with 10% FBS and 1% penicillin/streptomycin. MCF10DCIS.com were cultured in DMEM/F12 supplemented with 5% horse serum, 1.05 mM calcium chloride and 10 mM HEPES. ETCC-006 and ETCC-010 were cultured in RPMI supplemented with 10% FBS and 1% P/S. Cells were maintained at 37 °C with 5% CO_2_ and were mycoplasma-free.

### Antibodies

Western blotting and indirect immunofluorescence were performed using the following primary antibodies: β actin (A5441, Sigma-Aldrich, Ireland); β1 integrin (#1952, Merck Millipore, Ireland); cyclin B1 (#4135), estrogen receptor α (#8644), HER2 (#4290), EGFR (#4267), IGF-1R (#3027), pan AKT (#2920), pan ERK (#4696), pan p-AKT (#4060), pan p-ERK (#4377), p-EGFR (#3777), progesterone receptor A/B (#8757) [all from Cell Signaling Technology Europe, Leiden, Netherlands]; cyclin D1 (ab16663) and N-cadherin (ab19348) [both from Abcam, Cambridge, UK]; E-cadherin (#610181, BD Biosciences, San Jose, CA, USA); GAPDH (sc47724) and vimentin (sc32322) [both from Santa Cruz Biotechnology, Dallas, TX, USA]. Secondary antibodies for western blots were IRDye 800CW or 680RD (LI-COR Biosciences UK Ltd., Cambridge, UK); and the secondary antibody used for β actin indirect immunofluorescence was DyLight 488 donkey anti-mouse (Jackson ImmunoResearch Europe Ltd., Ely, UK).

### Hematoxylin and eosin staining

Cells were seeded onto sterile coverslips in 6-well plates and, after 24 h, were fixed with 4% paraformaldehyde (PFA) for 15 min. Cells were permeabilized with 0.2% triton X-100 in phosphate buffered saline (PBS) for 10 min and then placed in 10% methanol for 10 min. All samples were stained with Harris’ hematoxylin (Atom Scientific, UK) for 10 min, differentiated in 0.5% acid alcohol for 30 s, and blued in Scott’s Tap Water (1% (w/v) MgSO_4_, 0.06% (w/v) NaHCO_3_) for 1 min. The cells were then counterstained with eosin Y 1% Alcohol (GCC Diagnostics, UK) for 5 min and imaged using EVOS FL Auto Imaging System (Invitrogen, Biosciences Ltd., Ireland).

### Immunofluorescence

Cells were seeded in a 6-well plate onto sterile coverslips and allowed to grow for 48 h. Cells were then fixed with 4% paraformaldehyde (PFA) in PHEM buffer (60 mM PIPES, 25 mM HEPES, 10 mM EGTA, 2 mM MgCl_2_, pH 6.9), quenched with 50 mM ammonium chloride and permeabilized with 0.1% Triton X in PHEM buffer for 5 min. Non-specific antibody binding was blocked by incubating the coverslips with 5% goat serum/PHEM for 30 min. Cells were stained with anti-β actin (1:400) overnight at 4 °C in a humidified chamber and washed with PHEM. The coverslips were then incubated with donkey, anti-mouse, 488-fluorophore (1:1000) conjugated secondary antibody and DAPI (4′,6-diamidino-2-phenylindole) at room temperature for 1 h then washed with PHEM. The coverslips were mounted onto Fluoroshield histology mounting medium (Sigma-Aldrich, Ireland). Images were acquired using a Leica DM6000 upright fluorescence microscope.

### Western blotting

Cells were plated onto 10 cm tissue culture dishes and were harvested when reaching about 70% confluency. In one experiment, cells were either grown in complete media or serum starved for 24 h before harvesting. Cells were scraped from the plates and lysed using NP-40 lysis buffer (150 mM NaCl, 50 mM Tris HCl pH = 8.0, 1% NP-40 and 1X cOmplete EDTA-free Protease Inhibitor Cocktail (Sigma-Aldrich, Ireland) for 30 min on ice. Cleared lysates were prepared by centrifugation for 30 min at 12,000 g at 4 °C. Protein levels were quantified using Pierce Bicinchoninic Acid (BCA) Protein Assay Kit (Thermo Fisher Scientific, Biosciences Ltd., Ireland) and equal amounts of protein samples (40–50 μg) were resuspended in SDS loading buffer and electrophoresed on 10% SDS-PAGE (sodium dodecyl sulfate-polyacrylamide gel electrophoresis) at 100 V for 2 h. Proteins were transferred onto polyvinylidene difluoride (PVDF) membrane (Merck Millipore, Ireland) for 1 h at 100 V then blocked with 5% milk or bovine serum albumin (BSA) for 1 h at room temperature and incubated with the primary antibody overnight at 4 °C. The next day, the membrane was washed with Tris-buffered saline + 0.1% Tween-20 (TBST) for 5 min, 3 times, incubated with secondary antibody for 1 h at room temperature, washed with TBST for 5 min, 3 times, and visualized using an Odyssey Imaging System (LI-COR Biosciences).

### RNA analysis by quantitative, reverse transcriptase, polymerase chain reaction (qRT-PCR)

Total RNA was extracted from cells using TRIzol (Thermo Fisher Scientific). Briefly, 0.2 mL of chloroform was added per 1 ml of TRIzol reagent, samples were homogenized and then left at room temperature for 3 min. The aqueous phase was separated by centrifugation, and RNA was precipitated using isopropanol. After two washes using 75% ethanol, the RNA pellet was airdried, resuspended in water and incubated at 58 °C for 10 min.

1 μg of RNA was treated with DNase to eliminate contaminating DNA using TURBO DNase (Invitrogen, Biosciences Ltd., Ireland) then used in a cDNA synthesis reaction using Superscript II (Thermo Fisher Scientific, Biosciences Ltd., Ireland) as per manufacturer instructions. Reactions lacking reverse transcriptase enzyme were also run in the same condition as controls. The synthesized cDNA was diluted 1:5 and used in qRT-PCR reactions. Briefly, 25 ng cDNA was combined with SYBR Green JumpStart Taq ReadyMix (Sigma-Aldrich, Ireland) in 20 ul reactions and run using the following conditions:

- 95 °C 10 min
- 94 °C 30 s, 57 °C 45 s, 72 °C 1 min repeated 39 times
- 94 °C 30 s, 57 °C 45 s, 72 °C 15 min
- Melting curve stage

qRT-PCR was performed using the StepOnePlus™ Real-Time PCR System and StepOnePlus software (Applied Biosystems, Foster City, CA, USA). After analysis of the melting curve, results were normalized to the expression of ubiquitin C, *UBC* [19], using the ΔΔC_T_ method. Technical duplicates were done for each reaction, and three biologicals replicates were processed. qRT-PCR primers used for each RNA target are listed in Table 1.

**Table 1:** DNA primers for qRT-PCR analyses of gene expression

Target Gene	Forward primer	Reverse primer
*BIRC5*	ACCGCATCTCTACATTCAAG	CAAGTCTGGCTCGTTCTC
*CCNB1*	CCAAATCAGACAGATGGAAAT	GCCAAAGTATGTTGCTCGA
*ESR1*	CCACCAACCAGTGCACCATT	GTCTTTCCGTATCCCACCTTTC
*GSTM1*	CTATGATGTCCTTGACCTCCACCGTA	ATGTTCACGAAGGATAGTGGGTAGCT
*HER2*	TCCTGTGTGGACCTGGATGAC	CCAAAGACCACCCCCAAGA
*MK167*	CCACACTGTGTCGTCGTTTG	CCGTGCGCTTATCCATTCA
*MYBL2*	AAAACAGTGAGGAGGAAC	CAGGGAGGTCAAATTTAC
*STK15*	GGAGAGCTTAAAATTGCAGATTTT	GCTCCAGAGATCCTTCTCAT
*PR*	TACCCGCCCTATCTCAACTACCTG	GGACACCATAATGACAGCCTGATG
*UBC*	TCGAGAATGTCAAGGCAAAGATC	GAGTGGACTCTTTCTGGATGTTGTA

### RNA sequencing of ETCC-006 and ETCC-010 transcriptomes

Total RNA was extracted using TRIzol reagent according to the manufacturer’s instructions. The RNA integrity was measured using the RNA 6000 Nano Kit (Agilent). Libraries were prepared using the New England Biolabs (NEB) Ultra II Directional RNA Library Prep Kit for Illumina (NEB #E7760) and NEBNext Poly (A) mRNA Magnetic Isolation Module (NEB #E7490). Libraries were sequenced using the Illumina NextSeq 500 sequencer and read 85 bases from library inserts (Genomics Core Facility, EMBL). The bioinformatic analysis was performed using the Galaxy platform [20]. FASTQ reads were trimmed and filtered for quality and adapter using Trimmomatic [21] and aligned to the human reference genome (hg19) using RNA Star [22]. FeatureCounts was used to quantify gene expression [23] and differential expression analysis was performed with DESeq2 [24] using RNAseq datasets generated for ETCC-006 and ETCC-010 against an MCF-10A RNAseq dataset (Project: PRJEB2623; Sample Accession: SAMEA1484687; Experiment Accession: ERX016686; European Nucleotide Archive (ENA), EMBL-EBI). Overexpressed genes with *p*-value < 0.05 were selected for pathway-based analysis using ReactomePA [25].

### Comparison of ETCC-006 and ETCC-010 to MCF10A, MCF10DCIS.com, MCF7 and MDA-MB-231 RNAseq datasets

From previous work, we received permission to access the RNA-seq data in Klijn et al. (2015) from the Genentech Data Access Committee (DAC) [18, 26]. Data was retrieved from the EMBL-European Genome-Phenome Archive (EGA) servers under EGAD00001000725. For this comparison, we used RNAseq datasets from normal-like breast epithelial cells (MCF10A), ductal carcinoma in situ (MCF10DCIS.com) [9], MCF-7 and MDA-MB-231 cell lines, along with the ETCC-006 and ETCC-010 datasets from this study. The RNAseq data was then aligned to the latest human genome reference sequence assembly, GRCh38, as provided by the National Center for Biotechnology Information (NCBI), using Spliced Transcripts Alignment to a Reference (STAR) [22]. Next, HTSeq was used to perform read counts [27]. The counts for each cancer cell line were then used as input data for GOseq on the Galaxy platform [28, 29]. For each cell line, the input data had gene/rna name (column1: c1), counts for that cell line (c2), counts in MCF10A (c3), and if counts were higher in the breast cancer cell lines than MCF10A (c4) - True/False. Gene ontology analyses for the top ten biological processes were visualised in GOseq [28].

### Cell proliferation monitoring

MCF-10A, MCF-7, MDA-MB-231, MCF10DCIS.com, ETCC-006 and ETCC-010 cells were seeded at a density of 50,000 cells per 12-well plate in triplicate. Media was replaced every third day. Cells were trypsinized, resuspended into medium and counted every 24 h over a period of 6 days. Trypan blue was used to monitor the viability of the cells. Population doubling time was calculated (16) using values at day 2 and day 5 with the following formula:

$$ \mathrm{Population}\kern0.17em \mathrm{Boubling}\kern0.17em \mathrm{Time}=\frac{duration\times \log (2)}{\log \left( final\kern0.17em cell\kern0.17em number\right)-\log \left( initial\kern0.17em cell\kern0.17em number\right)}. $$

### Clonogenic assay

MCF-10A, MCF-7, MDA-MB-231, MCF10DCIS.com, ETCC-006 and ETCC-010 cells were seeded at a density of 2000 cells per six-well plate in triplicate. Media was replaced every third day. Cells were washed with PBS and fixed with 96% ethanol for 10 min, then stained with 0.05% crystal violet in 20% ethanol for 30 min. Excess crystal violet was removed by washing with sterile, distilled water and plates were dried. Staining was assessed using an Odyssey Imaging System and quantified using ImageJ software.

### Wound healing assay

MCF-10A, MCF-7, MDA-MB-231, MCF10DCIS.com, ETCC-006 and ETCC-010 cells were seeded on plates with two-well, culture inserts (#80209, Ibidi GmbH, Gräfelfing, Germany) the day before performing the assay to form a monolayer. Removal of the insert created a wound of 500 μm width. Wound closure was monitored 5 h, 9 h and 12 h after removal of the insert. Images were acquired using the EVOS FL Auto Imaging System (Invitrogen, Biosciences Ltd., Ireland). Wound width was quantified using the ImageJ software.

### Anchorage-independent growth

MCF-10A, MCF-7, MDA-MB-231, MCF10DCIS.com, ETCC-006 and ETCC-010 cells were seeded at a density of 300,000 cells in an agarose suspension (0.4% agarose) over an agarose underlay (0.8% agarose) for assessment of anchorage-independent growth. Media was changed every 5 days. After 3 weeks of growth, the agarose layers were stained with 1% crystal violet at 4 °C overnight and destained through sterile, distilled, water washes every 24 h until single colonies appeared. Staining was assessed using an Odyssey Imaging System and counted by hand.

## Results

### Morphologic characterization of the DCIS cell lines ETCC-006 and ETCC-010, compared to MCF10A, MCF7, MDA-MB-231 and MCF10DCIS.com

The primary goal of our work was to further characterize the recently developed ETCC-006 and ETCC-010 DCIS cell lines [14]. Before comparing the molecular details of those cell lines, we first examined overall cell morphology by comparing ETCC-006 and ETCC-010 to another DCIS cell line, MCF10DCIS.com, a normal-like breast epithelial cell line, MCF10A, and two breast cancer cell lines, MCF7 and MDA-MB-231.

**Fig. 1:**
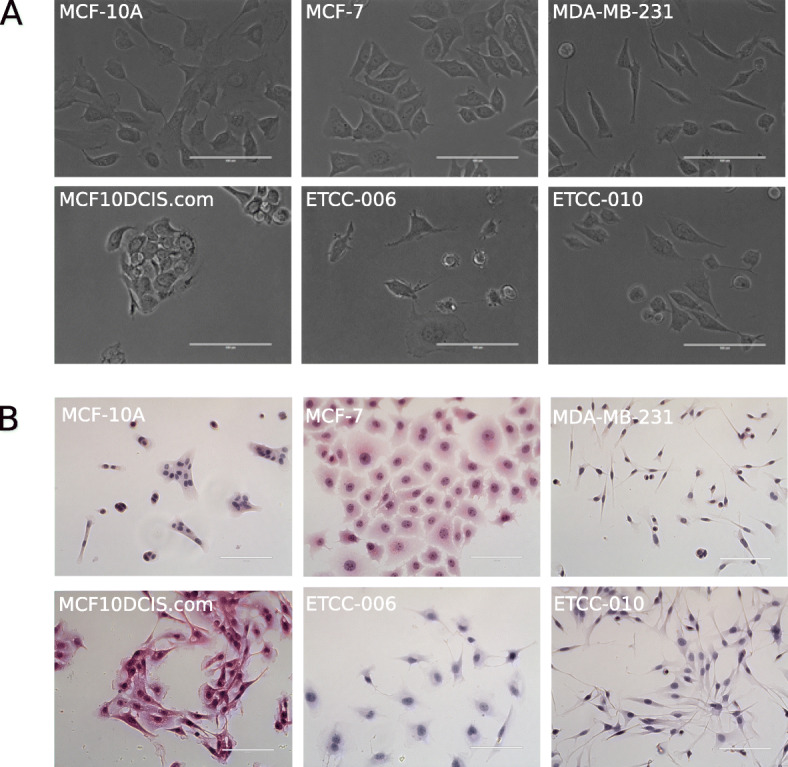
DCIS cell lines, MCF10DCIS.com, ETCC-006 and ETCC-010, show distinct cellular morphologies compared to other breast cancer cell lines and a normal-like breast epithelial cell line. Phenotypic and morphological analysis of MCF10DCIS.com, ETCC-006 and ETCC-010 in comparison with MCF-10A, MCF-7 and MDA-MB-231 cell lines by **A** bright field microscopy and by **B** hematoxylin and eosin (H&E) staining of fixed cells, followed by imaging using the EVOS FL Auto Imaging System at 40X magnification. Scale bar represents 100 μm

**Fig. 2:**
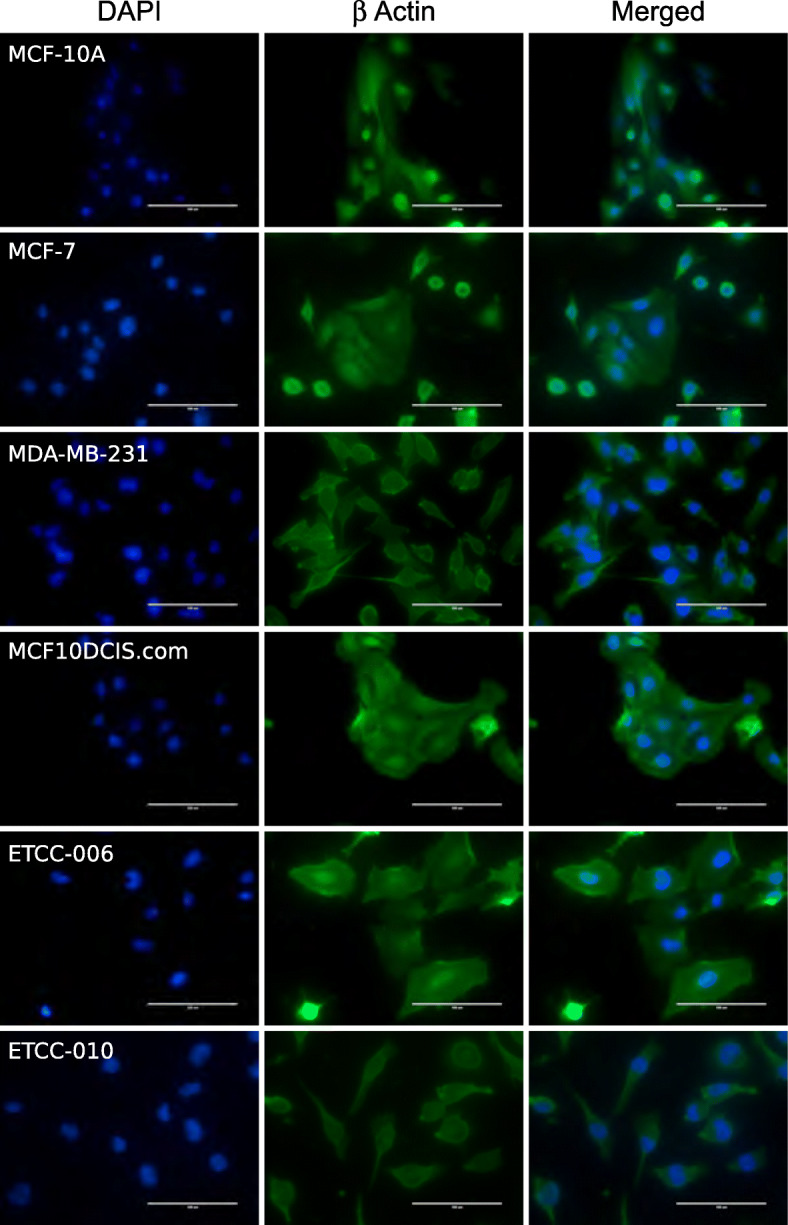
Cell morphologies of DCIS cell lines, MCF10DCIS.com, ETCC-006 and ETCC-010 compared to other breast cancer cell lines and a normal-like breast epithelial cell line as determined by immunofluorescence. Indirect immunofluorescence of fixed and permeabilized cells was performed using a primary antibody against β-actin and an anti-mouse Alexa Fluor 488 secondary antibody. DAPI was used to stain cell nuclei in the immunofluorescence analysis. Images were captured using the EVOS FL Auto Imaging System at 40X magnification. Scale bar represents 100 μm

Interestingly the two ETCC DCIS cell lines showed evident morphologic differences with each other, despite being isogenic and derived from the same tumor; yet, they displayed some striking similarities with the other cell lines examined. We studied their morphologic differences while growing the cells in a culture dish (Fig. 1A) and with cells grown on coverslips, followed by fixation and staining with hematoxylin and eosin (H & E staining) (Fig. 1B). Cells were also grown onto coverslips and fixed, prior to β-actin indirect immunofluorescence and DAPI staining of cell nuclei (Fig. 2). β-actin is a highly conserved and expressed protein, implicated in the control of cell growth and migration [30]. It is important for the maintenance of the cellular architecture; therefore, we chose it as an additional marker to visualize differences in cell morphology. In Figs. 1 and 2, differences in size, shape and organization can be noticed in the panel of cells examined.

Similar to MCF-7, MCF10DCIS.com cells displayed high cellular contact among cells, with cells forming clusters with each other. This suggests that those cells are highly dependent on cellular contacts for growth. Although cell size was not quantified, observation under the microscope suggests that MCF10DCIS.com cells are of similar size as MCF-7 cells.

Considering the ETCC lines, ETCC-006 cells seem to show the largest cell size of all cells analyzed in this study, with many cytoplasmic projections observed. Moreover, ETCC-006 cells were phenotypically striking because of their apparent flatness when visualized under the microscope; thus, observation under bright field microscopy proved to be more difficult than for other cell lines (Fig. 1A).

ETCC-010 cells were derived from the same DCIS tumor as ETCC-006, yet they show little morphologic similarities to each other. Instead, ETCC-010 cells displayed a high degree of resemblance to MDA-MB-231 cells (Figs. 1B and 2). ETCC-010 cells were elongated, with the nucleus occupying most of the available space within the cell. The difference in morphology between ETCC-006 and ETCC-010 is interesting since they both have the same genetic background, highlighting tumor heterogeneity within DCIS lesions [31].

### Molecular subtype of DCIS cell lines

**Fig. 3:**
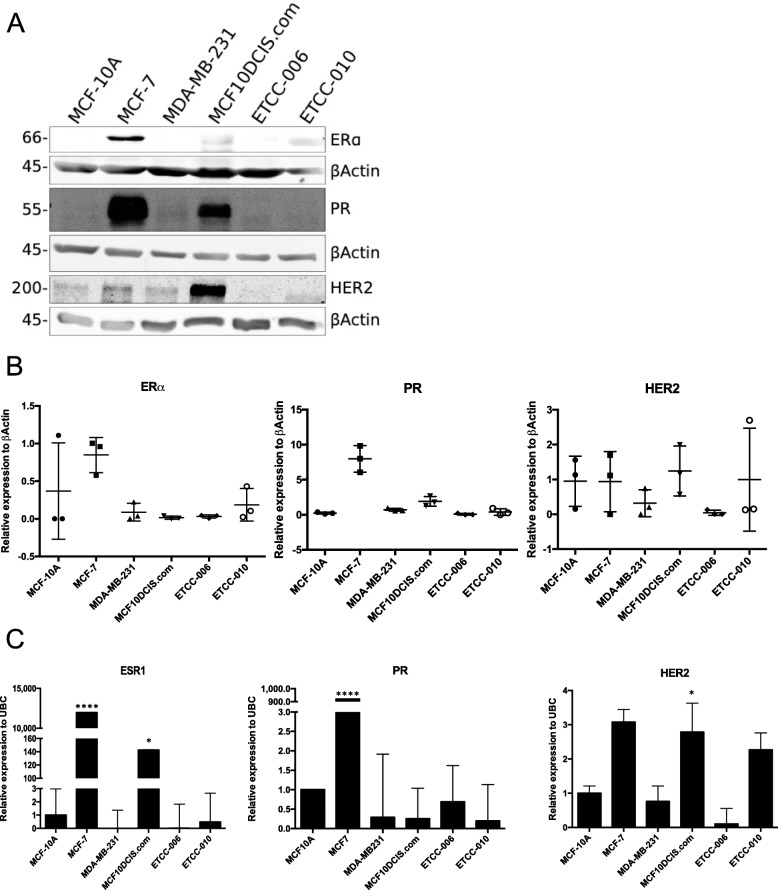
Molecular subtype based on hormone-receptor status of DCIS cell lines, ETCC-006 and ETCC-010. Estrogen receptor alpha (ERα), progesterone receptor (PR) and receptor tyrosine kinase ErbB-2 (HER2) levels were measured at the protein level (**A**) and (**B**) and RNA level (**C**) by immunoblotting and qRT-PCR. For the immunoblot, 42 μg of total protein was loaded per lane onto the gel. Protein level was quantified and normalized against βactin using ImageJ. For quantification in panel **B**, the horizontal bar indicates the mean with +/−standard error of mean (SEM). This experiment was repeated in triplicate and shown is a representative immunoblot. Full-length blots are presented in Supplementary Materials

In breast cancer care, testing of the ER, PR and HER2 receptor status is routinely done to assess the prognosis and the treatment that will be given to the patient [1]. In breast cancer research, the ER, PR and HER2 receptor status of cell lines is verified to determine which breast cancer subtype those cell lines represent. Before analyzing the ER, PR and HER2 receptor status of the DCIS cell lines, we confirmed the published molecular subtype of the control cell lines by immunoblot analysis. MCF-10A and MDA-MB-231 are known to have no expression of ER, PR and HER2. MCF-7 is a hormone receptor-positive breast cancer cell line, so it expresses ER and PR and modest levels HER2 [4] Those results were confirmed at both protein and RNA levels (Fig. 3).

MCF10DCIS.com has the same genetic background as MCF-10AT, which was derived from MCF-10A cells through oncogenic transformation with an activated *HRAS* gene [8, 32]; therefore, it is not surprising that similar to MCF-10A, this cell line shows little expression of ER at the protein and RNA level. PR was detected at the protein level (Fig. 3A and B) but not at the RNA level (Fig. 3C). Since levels of ER and PR in MCF10DCIS.com are low, those receptors are likely not functionally relevant. High levels of HER2 were detected in MCF10DCIS.com, explained by the presence of two gene copies of *ERBB2* [33, 34].

While ETCC-006 showed no expression of ER, PR and HER2, we detected very low levels of ER and no expression of PR and HER2 at protein and RNA level in ETCC-010 cells. These results contradict the previous work by Yong et al. (2014) [14]; however, our work shows directly that HER2 is not expressed in these two DCIS cell lines and agrees with previous work showing that treatment with Herceptin (monoclonal antibody directed against HER2) was not cytotoxic to ETCC cell lines [14]. Taken together, our work indicates that ETCC-006 and ETCC-010 are functionally similar, based on receptor status, to triple-negative breast cancer cell lines.

### Analysis of the gene expression of Oncotype DX DCIS markers in DCIS cell lines

Since we found that ETCC-006 and ETCC-010 display different characteristics to what was published by Yong et al. (2014) [14], we decided to characterize the cell lines using previously suggested DCIS markers. However, defining DCIS using a small set of known markers has been difficult [35], mainly because medical research on DCIS is more limited compared to that of IDC.

The Oncotype DX DCIS score is a 12-gene panel that generates predicted 10-year risk of local DCIS and invasive recurrence following treatment by breast conserving surgery [36]. It includes five gene markers of proliferation – *MKI67* (Ki67), *STK15* (AURKA), *BIRC5* (survivin), *CCNB1* (cyclin B1), *MYBL2* (MYB proto-oncogene like 2); *PGR* (progesterone receptor), *GSTM1* (glutathione S-transferase M1) and five reference genes. While it is not relevant to calculate a DCIS score for cell lines, we decided to assess the expression levels of those markers in the DCIS cell lines, ETCC-006 and ETCC-010, compared to the other cell lines in our panel.

Supplemental Figure 1 shows the results of qRT-PCR to analyse the expression of *MKI67*, *STK15*, *BIRC5*, *CCNB1*, *MYBL2*, *GSTM1* and *PGR*. MCF-7 cells show high expression of the proliferation markers (Panel A) along with high expression of *GSTM1* and *PGR* (Panel B). MDA-MB-231 cells show high expression of four proliferation markers, very low expression of *PGR* and reduced *GSTM1* expression compared to MCF-7 cells. Regarding MCF10DCIS.com, we observed lower expression of the proliferation markers, when compared to the breast cancer cell lines, and low *GSTM1* and *PGR* expression. It is important to note that while *PGR* RNA levels appeared low as determined by qRT-PCR; PR protein was detectable by immunoblotting (Fig. 3). Meanwhile, ETCC-006 showed high expression of four proliferative markers and low expression of *PGR* and *GSTM1*. ETCC-010 displayed a similar trend to MCF10DCIS.com cells, with comparable levels of *MKI67*, *STK15* and *BIRC5* expression. However, ETCC-010 showed slightly higher expression of *GSTM1* and lower expression of *PGR*, compared to ETCC-006. Analysis of this small panel of genes suggests that differences in gene expression observed between the ETCC DCIS lines likely reflects the cellular heterogeneity of the original tumor.

### Genome-wide comparison of the ETCC-006 and ETCC-010 DCIS model to MCF-10A

In parallel to analyzing the molecular subtype of our cells, we performed RNA sequencing (RNAseq) of ETCC-006 and ETCC-010 cell lines. RNAseq allows the identification of transcripts present in the ETCC-006 and ETCC-010 cells at a genome-wide level. By comparing the level of expression of transcripts in ETCC-006 and ETCC-010 to those in MCF-10A cells, we aimed to identify if genes in some cancer-related pathways showed altered expression. Before performing RNAseq, we analyzed the quality of the total RNA extracted from our cell lines using bioanalyzer traces (Agilent) to calculate an RNA integrity (RIN) value that is indicative of the level of degradation of the RNA. RIN values range from 0 (degraded) to 10 (intact). We obtained RIN values of 10 for RNA extracted from ETCC-006 and ETCC-010, each with two replicates. This indicated that the RNA was of good quality and could be used for library preparation.

**Fig. 4:**
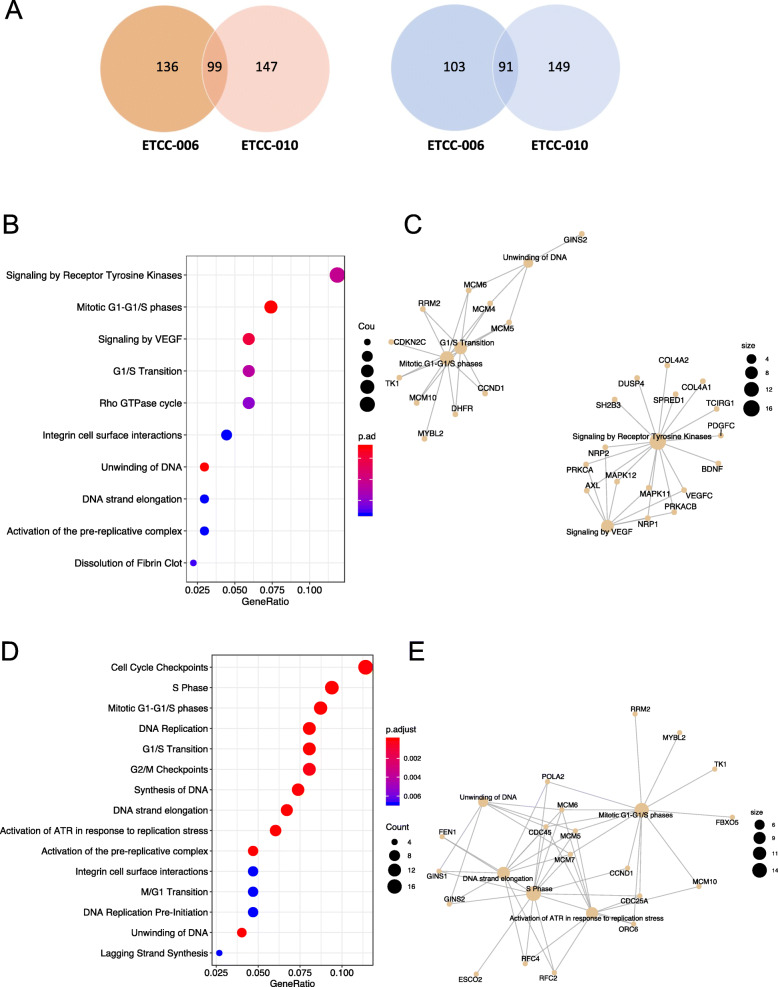
Transcriptomic comparison of the DCIS cell lines ETCC-006 and ETCC-010 to MCF-10A. **A** Venn diagram of the number of genes with higher expression in orange and lower expression in blue in ETCC-006 and ETCC-010 compared to MCF-10A (*p*-value < 0.05). **B** ETCC-006 reactome-pathway analysis and **D** ETCC-010 reactome-pathway analysis; **C** ETCC-006 and **E** ETCC-010 associated String pathway analyses of the differentially over-expressed genes in ETCC-006 and ETCC-0010 (*p*-value < 0.05) to identify enriched pathways in those cells and the genes associated with those pathways

After RNA sequencing, reads were trimmed and filtered for quality with Trimmomatic [21] and aligned to the human reference genome (hg19) with RNA STAR [22]. Gene expression was quantified with FeatureCounts [23] and differential expression was performed using DESeq2 [24]. We compared the transcriptome of the ETCC lines to that of MCF-10A, using an available dataset, as detailed in the Methods. After the differential expression, we excluded genes with a *p*-value over 0.05. Figure 4A is a Venn diagram of the number of genes with higher expression in ETCC-006 and ETCC-010 in orange and lower expression in ETCC-006 and ETCC-010 in blue. In total, 235 and 246 genes were over-expressed in ETCC-006 and ETCC-010, with 99 genes in common; while 194 and 240 genes were under-expressed in ETCC-006 and ETCC-010, with 91 genes in common (Fig. 4A). All genes identified from our RNAseq analysis are listed in Additional file 1.

**Fig. 5:**
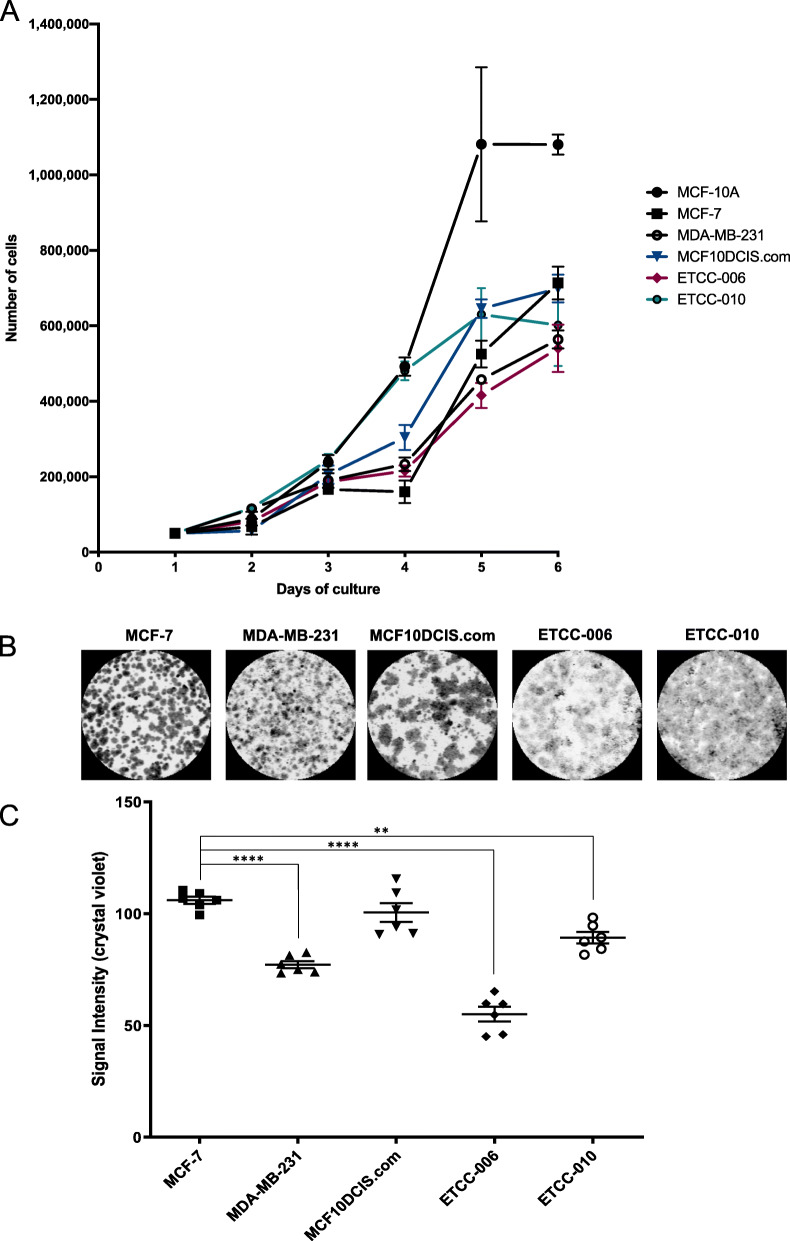
Proliferation and survival assessment of the DCIS cell lines. Cell proliferation was monitored by trypan blue staining over six days (**A**); while cell survival was assessed by a clonogenic assay, followed by crystal violet staining (**B**) and quantification using ImageJ (**C**). Statistical analysis of the clonogenic assay was done using one-way ANOVA, with **** *p*-value < 0.0001; ** *p*-value < 0.01. Horizontal bars show the mean signal intensities +/− SEM (GraphPad Prism v.8.3.0)

Next, reactome pathway-based analyses were performed using an R/Bioconductor package, ReactomePA [25], using the lists of over-expressed genes in the ETCC DCIS cell lines to identify enriched pathways (Fig. 4B and D). The most enriched pathways common to ETCC-006 and ETCC-010 related to cell proliferation: mitotic G1-G1/S phases, G1/S transition, unwinding of DNA (S phase), etc. Interestingly, integrin-cell surface interaction gene products were enriched in both cell lines compared to MCF-10A. Since those regulate cell motility, it possibly indicates that ETCC-006 and ETCC-010 have migratory abilities. ReactomePA also allowed us to generate String analyses of the genes implicated in the pathways mentioned (Fig. 4C and E). Proliferation in both cell lines seems to rely mostly on commonly upregulated genes (*CCND1*, *MCM* family, *MYBL2*, etc.). CCND1 is an important regulator of the G1/S transition [37], the MCM proteins constitute a family of protein involved in the regulation of DNA replication [38], and MYBL2 is a transcription factor involved in the regulation of the G1/S transition [39]. Interestingly, an enrichment of “signalling by VEGF” was observed in ETCC-006 (Fig. 5B), and VEGF (vascular endothelial growth factor) signalling has been shown to be upregulated in DCIS [40]. Overall, most enriched pathways in ETCC-006 and ETCC-010 are related to DNA replication and proliferation.

### Gene ontology analyses of ETCC-006 and ETCC-010 compared to MCF10A, MCF10DCIS.com, MCF7 and MDA-MB-231

To expand our comparison of ETCC DCIS RNAseq data, we decided to analyze the ETCC-006 and ETCC-010 datasets to RNAseq data from MCF10A, MCF10DCIS.com, MCF7 and MDA-MB-231 cell lines from Klijn et al. (2015) [18]. This dataset is particularly useful due to its inclusion of the MCF10DCIS.com cell line, as this cell line is not present in other breast cancer cell line RNAseq datasets [41]. For this analysis, RNAseq data was aligned to the latest human genome reference sequence, GRCh38, and gene ontology analysis was performed using GOseq [28]. GOseq is an application specifically designed for performing gene ontology (GO) analysis on RNAseq data and has the advantage of eliminating biased results due to overrepresentation of long and highly expressed transcripts [28]. In Supplemental Figure 2, panels for ETCC-006 and ETCC-010 are shown at the top; while panels for MCF10DCIS.com, MCF7 and MDA-MB-231 cells are below. All cell lines had distinct GO profiles, with ETCC-006 showing enrichment of gene sets involved in extracellular organization and ETCC-010 with ion transport. For ETCC-010, we found the link to ion transport intriguing, as DCIS in patients is often diagnosed by the presence of microcalcifications in radiographs [42]. Similar to our previous observations, these two isogenic DCIS cell lines are distinct from each other and different to the previously characterised MCF10DCIS.com line [9]. Meanwhile from our analysis of RNAseq data from MCF7 cells, gene sets involved in transcription and RNA regulation were enriched; whereas, MDA-MB-231 cells displayed differential expression of genes linked to metabolism (Supplemental Figure 2).

Although our pathway analysis and GO enrichment of gene sets are distinct, we did find overlap in ETCC-006 cells between the *COL4A1/A2* (collagen type IV) genes within the receptor tyrosine kinase STRING analysis (Fig. 4C) and the GO analysis (Supplementary Figure 2), in which extracellular organization genes were enriched. Since type IV collagens are major components of the basement membrane [43] and their overexpression is linked to cancers of the brain, stomach and liver [44–46], expression and/or modulation of *COL4A1/A2* genes might be part of the molecular changes associated with DCIS.

### Monitoring of cell proliferation and survival in DCIS cell lines, ETCC-006 and ETCC-010

To confirm the observation from the pathway enrichment analysis that proliferation is promoted in the ETCC lines, cell growth was monitored to determine population doubling time (PDT) using trypan blue staining. Figure 5A shows proliferation curves for each of the cell lines examined. MCF-10A, MCF-7 and MDA-MB-231 showed different rates of proliferation with PDT of 20 h, 24.4 h and 36.2 h respectively. The rate of proliferation of MCF10DCIS.com was comparable to that of MCF-10A, with 20.5 h PDT. ETCC-006 and ETCC-010 showed intermediate proliferative rates to that of MCF-7 and MDA-MB-231, with 30.7 h and 29.7 h respectively. Interestingly, while most cell lines seemed to undergo a lag phase after plating, the ETCC-010 growth profile displayed high proliferative abilities even at low cell numbers (Fig. 5A).

To extend our characterization of the ETCC cell lines, we used the clonogenic assay to monitor cell survival by testing the cells’ ability to go through cell division with no limit in time [47, 48]. The clonogenic assay reflects cell survival in vitro by measuring the staining intensity of colonies formed with crystal violet. Following growth and replenishing cultures with fresh media, cells were fixed after 10 days and colonies were stained (Fig. 5B). Compared to MCF7 cells, MDA-MB-231 and ETCC-006 cells showed reduced colony formation, which was quantitated by crystal violet staining intensity (Fig. 5C). For ETCC-006, the reduction in signal intensity might be explained by the characteristic flatness of the cells that makes them unsuitable for crystal violet staining. Interestingly, while MCF-7 and MDA-MB-231 showed distinct colonies, all DCIS cell lines appeared more like a monolayer, with an observable reduction in colony formation particularly for the ETCC DCIS lines (Fig. 5C).

### Assessment of migration and anchorage-independent growth potential of the DCIS cell lines

**Fig. 6:**
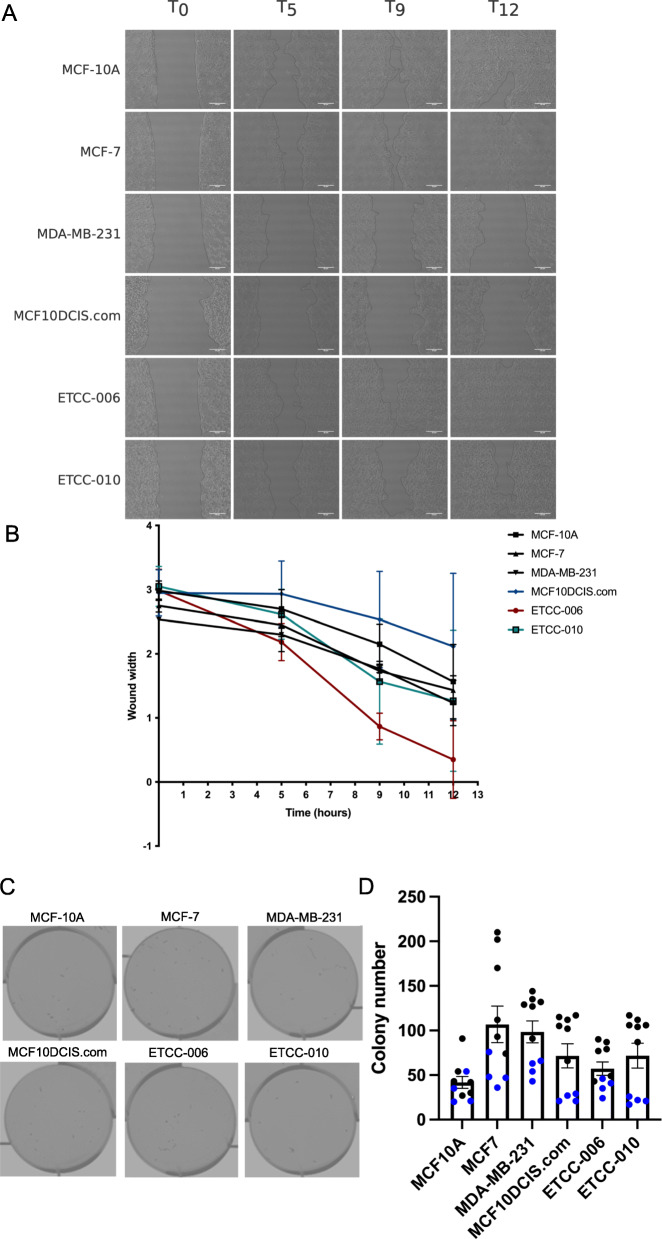
Migration and anchorage-independent growth assessment of the DCIS cell lines. Migratory capacity of the DCIS cell lines, in comparison to MCF10, MCF7 and MDA-MB-231 cells, was examined by a wound healing assay. The wound was imaged using bright field microscopy at 20X (**A**) and pseudo-marked in black. **B** wound width was quantified in ImageJ in arbitrary units (*N* = 3). Anchorage-independent growth was measured by soft agar assays; wells were imaged using the Odyssey Li-Cor system (**D**) and scored manually (**E**). Shown in panel E are two biological replicates (black/blue), with each technical replicate plotted. Horizontal bars show the mean number of colonies formed +/− SEM (GraphPad Prism v.8.3.0)

Since the integrin cell surface interactions pathway was enriched in ETCC-006 and ETCC-010 (Fig. 4B and D) and the GO analysis indicated gene set enrichment in extracellular structure organization (Supplemental Figure 2), we monitored the migratory abilities of the DCIS cell lines using a wound healing assay. Wound width and wound area was examined after removal of the culture insert at time zero hours (T0), 5 h after insert removal (T5), and then at nine and 12 h post-insert removal (T9 and T12). Using bright-field microscopy, we observed wound closure within 12 h in MCF-10A, MCF-7 and ETCC-006 cells (Fig. 6A - C). In ETCC-010, after 12 h the wound was close to closure. MCF10DCIS.com and MDA-MB-231 showed slower wound closure, a wound measurement over still over 100 μm after 12 h.

Anchorage-independent growth, a property of most malignant cells to grow independently of a solid surface, was evaluated by a soft agar colony formation assay [49]. As expected, breast cancer cell lines, MCF-7 and MDA-MB-231, showed the highest ability to form colonies in soft agar. The three DCIS cell lines MCF10DCIS.com, ETCC-006 and ETCC-010 showed similar abilities to form colonies in agar, but with numbers intermediate to MCF-10A (lowest) and MCF-7 or MDA-MB231 (highest) (Fig. 6D and E).

### ETCC-006 and ETCC-010 DCIS cell lines express markers of epithelial-mesenchymal transition

**Fig. 7:**
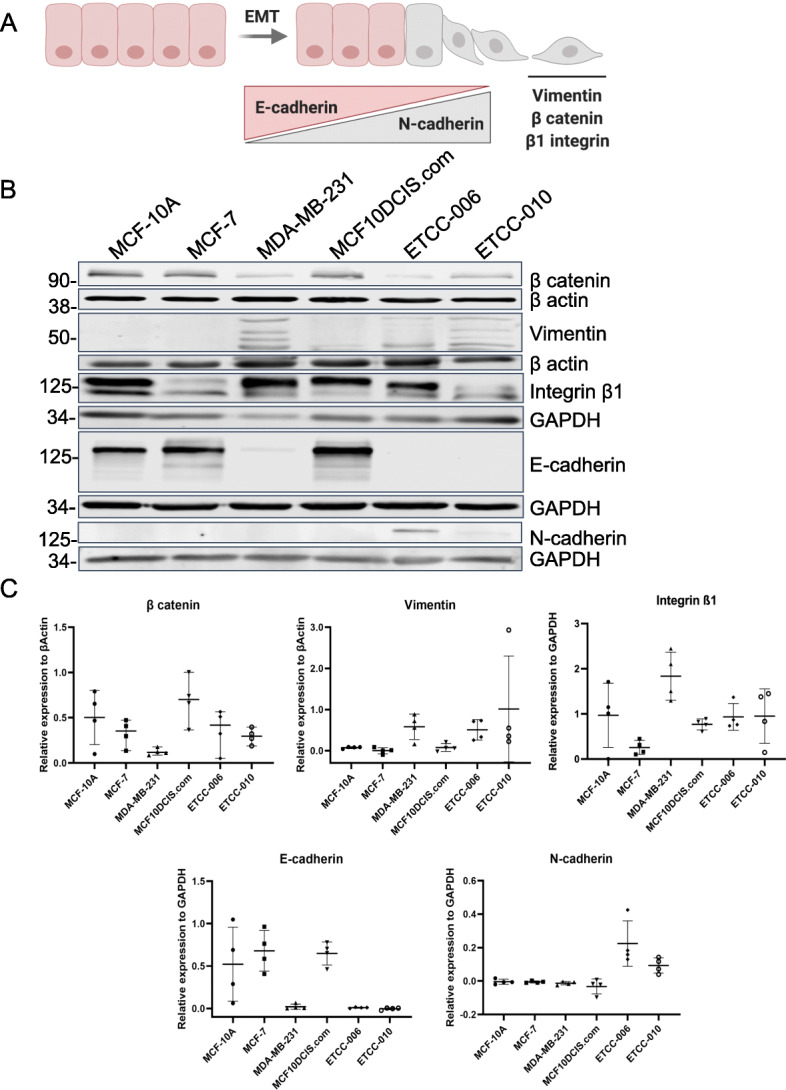
ETCC-006 and ETCC-010 display markers of epithelial to mesenchymal (EMT) transition. **A** Schematic illustrating the markers associated with EMT **B** Western blot analysis of EMT markers in the DCIS cell lines compared to breast cancer cell lines, MCF7 and MDA-MB-231, and normal-like breast epithelial cells, MCF10A. 40 μg of total protein was loaded per lane onto the gel. **C** Protein level was quantified and normalized against β actin or GAPDH using ImageJ. This experiment was repeated with four, independent biological replicates (*N* = 4), panel **B** is a representative immunoblot. Panel **A** was created with BioRender.com. Full-length blots are presented in Supplementary Materials

Taken together, our data has shown that the DCIS cell lines ETCC-006 and ETCC-010 show enrichment in migration pathways (Fig. 4), extracellular organization genes (Supplemental Figure 2) and have migration abilities (Fig. 6). Next we sought to consider whether those cell lines have undergone epithelial to mesenchymal transition (EMT). EMT is a common event in cancer progression where epithelial cells lose their epithelial phenotype, characterized by tight and adherent cells, to gain a mesenchymal phenotype (Fig. 7A). Moreover, during EMT, cells lose expression of epithelial markers, such as E-cadherin, to gain expression of mesenchymal markers (vimentin, N-cadherin, etc.). Along with cell motility [50], activation of EMT programs contribute to the events leading to metastasis [50–52].

To examine the level of expression of key EMT markers, whole cell lysates from each cell line were prepared. Vimentin, E-cadherin, N-cadherin, β-catenin and β1-integrin expression was analyzed by immunoblotting. High expression of vimentin was observed in ETCC-006 and ETCC-010 at similar levels to those observed in MDA-MB-231; while low expression of vimentin was detected in MCF10DCIS.com at level comparable to MCF-10A (Fig. 7B and C).

Most strikingly, while MCF10DCIS.com cells expressed E-cadherin, we could not detect expression of E-cadherin in ETCC-006 and ETCC-010. Instead, expression of N-cadherin was observed in ETCC-006 and ETCC-010 lysates. β-catenin is a transcription factor induced by the WNT pathway that stimulates transcription of genes involved in the EMT [53], and its expression is associated with poor prognosis in breast cancer [54]. In our analysis, β-catenin protein levels were elevated in MCF-10A, MCF10DCIS.com and ETCC-006, while levels were more balanced in MCF-7, MDA-MB-231 and ETCC-010 cells. β1-integrin is a transmembrane protein, whose function is to bind extracellular matrix (ECM) [55] and regulate a cell’s ability to invade [56]. β1-integrin was detected at high levels in MCF-10A, MDA-MB-231 and ETCC-006, with ETCC-010, MCF10DCIS.com and MCF-7 showing lower protein levels.

Overall, the co-expression of β-catenin, vimentin, β1 integrin and N-cadherin suggests that ETCC-006 and ETCC-010 have undergone an EMT transition. Although MCF10DCIS.com cells express some markers of EMT, overall it is more representative of cells with an epithelial phenotype.

### Signalling profile and cell cycle proteins in DCIS cell lines

**Fig. 8:**
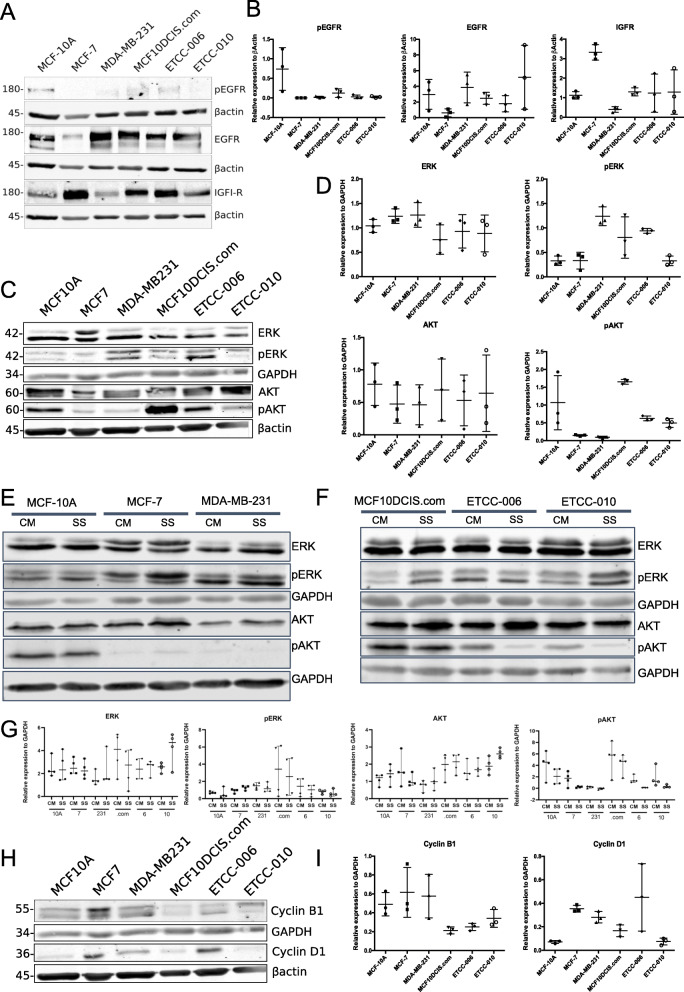
Profile of cell signalling and cell cycle proteins in the DCIS cell lines compared to breast cancer cell lines. **A** and **B** IGF-1R, EGFR and p-EGFR protein level. **C** ERK, p-ERK, AKT and p-AKT protein levels under normal conditions. **D**, **E** and **F** Comparison of ERK, p-ERK, AKT and p-AKT protein levels under normal conditions and serum-starvation conditions. **H** and **I** Cyclin B1 and D1 protein levels. Protein level was quantified and normalized against β actin or GAPDH using ImageJ. Experiments were repeated in triplicate, except the serum-starvation experiments were repeated with four, independent biological replicates. Horizontal bars in panels **B**, **D**, **G** and **I** indicate the mean protein expression relative to the loading control +/− SEM (GraphPad Prism v.8.3.0). Panels **A**, **C**, **E**, **F** and **H** are representative immunoblots. Full-length blots are presented in Supplementary Materials

Since signalling by receptor tyrosine kinases was most enhanced in ETCC-006 cells according to our RNAseq data and ReactomePA analysis (Fig. 4), and ETCC-006 and ETCC-010 have undergone EMT (Fig. 7), we next decided to investigate levels of some of proteins in major cell signalling pathways. We measured protein levels of two receptor tyrosine kinases, Epidermal Growth Factor Receptor (EGFR) and Insulin-like Growth Factor 1 Receptor (IGF-1R), both critical in the processes leading to metastasis in breast cancer [57, 58]. To do this, cell lysates were prepared using cells grown in normal conditions. Total levels of IGF-1R, EGFR and the active phosphorylated-EGFR were examined by immunoblotting (Fig. 8A and B). Low protein levels of EGFR were detected in MCF-7, with the other cell lines showing similar levels of EGFR. In MCF-10A cells, phosphorylation of EGFR was detected; whereas, p-EGFR was not detected in other cell lines under normal conditions. IGF-1R showed high levels in MCF-7 and low levels in MDA-MB-231 cells. The DCIS cell lines showed similar levels of IGF-1R, with intermediate levels to those observed in MDA-MB-231 and MCF-7 cells.

Common downstream targets of EGFR and IGF-1R include the Ras/Raf/MEK/ERK and PI3K/ AKT pathways, known to stimulate proliferation, migration and EMT when activated after being phosphorylated [53]. While total levels of ERK were similar across DCIS cell lines (Fig. 8C and D, normalized to GAPDH protein), phosphorylated ERK (p-ERK) levels seem slightly higher in MDA-MB-231, MCF10DCIS.com and ETCC-006. Similarly, total AKT levels were similar across cell lines; however, phosphorylated AKT (p-AKT) showed high disparity (normalized to β-actin). Low p-AKT was observed in MCF-7 and MDA-MB-231 cells. Higher levels of p-AKT were observed in MCF-10A, ETCC-006 and ETCC-010, while MCF10DCIS.com showed consistently high levels of p-AKT.

Next, we aimed to investigate whether AKT is constitutively activated in MCF10DCIS.com cells. To do this, we compared the phosphorylation levels of AKT under normal and serum starvation conditions (Fig. 8E, F and G). In MCF7, MDA-MB-231, ETCC-006 and ETCC-010 cells, we found that p-AKT is lost after serum starvation conditions. We confirmed the constitutive activation of AKT in MCF10DCIS.com cells, as p-AKT levels remain unaffected under serum starvation conditions. This was also observed in MCF10A cells (Fig. 8E) and could reflect variability in PI3K levels [59].

Since high levels of cyclin D1 are associated with DCIS [60], we examined the levels of cyclin B1 and cyclin D1, two regulatory proteins in the cell cycle (Fig. 8H and I). While the cyclin B1 level seemed slightly lower in the DCIS cell lines compared to the control cell lines, cyclin D1 showed high expression in MCF-7 and ETCC-006, in agreement with our RNAseq results (Fig. 4).

## Discussion

Choosing the correct model, animal or cellular, is critical in research. To study DCIS in an in vitro cell model, we choose to use three DCIS cell lines: MCF10DCIS.com, a well characterized DCIS cell line model, and ETCC-006 and ETCC-010, two patient-derived cell lines. To date, other research groups have not used these recently established patient-derived lines; therefore, we conducted a baseline phenotypic and molecular characterization of DCIS cell lines, ETCC-006 and ETCC-010.

Despite some differences in in vitro and in vivo assays as highlighted in the original publication, ETCC-006 and ETCC-010 share the same genetic background. For many of the properties examined in this work, we mostly found that ETCC lines behave quite similar to each other in vitro, with similar rates of proliferation, colony formation and anchorage-independent growth. Still the two lines have very different transcriptional outputs, with less than 30% of differentially expressed transcripts being common between the two cell lines. Similarly, ETCC-006 shows more enrichment of proliferative markers than ETCC-010, with cyclin D1 more highly expressed in ETCC-006 compared to ETCC-010 in our RNAseq data (Additional file 1) and at protein levels (Fig. 8H and I). A limitation of our RNAseq analysis is that both ETCC lines were compared to an existing dataset from MCF-10A cells that do not share the same genetic background. However, we believe that MCF-10A RNAseq data is the best choice for a comparison, and this is the first transcriptomic study of the ETCC DCIS cell lines. This was extended with our GO analysis using existing RNAseq datasets from Klijn et al. (2015) [18] to compare the ETCC DCIS cell lines to MCF10DCIS.com, MCF7 and MDA-MB-231 breast cancer cell lines, again using MCF10A cells as the ‘baseline’ transcriptomic profile (Supplemental Figure 2).

In one key finding, we showed that ETCC-006 and ETCC-010 are more similar to triple-negative breast cancer cell lines, with no expression or limited expression of ER, PR and HER2 (Fig. 3). This is contrary to what was described in the original publication [14]. Importantly, we have concerns regarding the original classification of ETCC cells as ER+, as the immunoblot using MCF7 cells, widely considered as an ERα-positive “prototype”, did not show ERα protein expression [14]. We did consider that expression of ER and PR could be lost over time; however, at the time of our study, we started with cells on passage 15 for ETCC-006 and 19 for ETCC-010 and purchased directly from the cell line repository DMSZ at the Leibniz Institute. Additionally, it has been argued that loss of steroid receptor expression by cells in culture is unlikely [61].

One of our most interesting observations was that ETCC-006 and ETCC-010 cells express markers of EMT (vimentin, N-cadherin and β1 integrin) (Fig. 7), suggesting that they have undergone a transition to a more mesenchymal-like phenotype. While they showed comparable proliferation abilities to other cell lines examined, both ETCC lines displayed greater migratory capacity compared to MCF10DCIS.com in a wound closure assay. Taken together, this suggests that ETCC-006 and ETCC-010 may represent models of high-grade DCIS.

Throughout this work, we have compared ETCC-006 and ETCC-010 to MCF10DCIS.com cells, a well-characterized DCIS cell line that produces comedo DCIS lesions in xenografts [9, 12].

Compared to the ETCC lines, MCF10DCIS.com appears to have conserved an epithelial phenotype with cells showing tight contact, no expression of EMT markers and limited migratory abilities. We did show constitutive activation of AKT, likely explained by a mutation in the PI3K gene [62, 63]. Indeed, MCF10DCIS.com has been shown to have a gain of function mutation, H1047R, in the catalytic domain of PI3K [62]. This mutation is one of the most oncogenic mutations and occurs frequently in other types of cancer [64]. Despite this activating mutation of the PI3K/AKT pathway, taken together our data suggests that the MCF10DCIS.com cell line may be less aggressive than the ETCC lines.

Where do DCIS cell lines fit in terms of the clinical representation of DCIS? As previously described, DCIS is an early stage of breast cancer, with a large spectrum of cases classified from low-grade disease to high-grade DCIS. High-grade DCIS is associated with a higher risk of developing IDC [65]; therefore if the ETCC lines truly represent high-grade DCIS, they could be a valuable tool to study the mechanisms leading to the transition between DCIS and IDC. In addition to ETCC-006 and ETCC-010, four more cell lines were derived from the same DCIS lesion, ETCC-001, ETCC-007, ETCC-008 and ETCC-011 [14]. It would be interesting to include these lines for future studies and further characterization. However, the overall number of DCIS cell lines is still limited, highlighting an ongoing need for more DCIS cell lines to be established and characterized, as pointed out recently by Brock et al. [13].

In the clinical DCIS spectrum, MCF10DCIS.com cells have been characterized as a model of human comedo DCIS in xenografts [9, 12]. In terms of histopathology, comedo DCIS is considered high-grade DCIS [66]. While we were unable to compare what type of tumor is induced by MCF10DCIS.com, ETCC-006 and ETCC-010 when injected in mice, our study does suggest that MCF10DCIS.com is an in vitro model of DCIS that is less aggressive than the ETCC lines. Together with established DCIS lines, we propose that continued use of the ETCC lines in xenografts, 2D co-culture and 3D culture systems (reviewed by Brock et al. [13]) may facilitate the search for the specific molecular requirements that permit breast cancer cells to transition from DCIS to IDC.

## Conclusions

Patient-derived DCIS cell lines, ETCC-006 and ETCC-010, exhibit molecular markers and cellular behaviour consistent with a more aggressive form of DCIS. Although both lines were derived from the same DCIS lesion, they display markedly different transcriptomic profiles, supporting intra-tumoral heterogeneity within DCIS affected areas of the breast. Our results provide a baseline phenotypic and molecular characterization of these newly established DCIS cell lines for further exploration by the research community.

## Supplementary Information

**Additional file 1. **Differentially expressed genes in ETCC-006 and ETCC-010 cell lines compared to MCF10A cells as determined by RNAseq. Genes that are over- and under-expressed and are unique to ETCC-006 and ETCC-010 cell lines are listed in separate sheets (ETCC-006_up; ETCC-006_down; ETCC-010_up; ETCC-010_down), as well as genes common between the two lines (Common_up and Common_down). Included in the files are the Ensembl gene identifiers, gene name and differential expression criteria, most notably log_2_(FC) and *p*-value. Only genes with a *p*-value < 0.05 are included.

**Additional file 2: Supplemental Figure 1.** Expression level of Oncotype DX DCIS score markers in the DCIS cell lines analyzed by qRT-PCR. (A) Relative expression of the proliferation markers is associated with an increased risk of DCIS recurrence whereas (B) *GSTM1* and *PGR* have a protective role against the DCIS risk of recurrence. qRT-PCR was performed using cDNA synthesized from total RNA isolated from normal-like and breast cancer cell lines, MCF-10A, MCF-7, MDA-MB-231, and DCIS cell lines, MCF10DCIS.com, ETCC-006 and ETCC-010. All data was normalized to *UBC* (polyubiquitin-C) and statistical analysis was done using one-way ANOVA, with **** *p*-value < 0.0001; *** *p*-value < 0.001; ** *p*-value < 0.01; * *p*-value < 0.05. Horizontal bars show the mean +/− SEM (GraphPad Prism v.8.3.0).

**Additional file 3: Supplemental Figure 2.** GO analysis from the differential expression of RNA transcripts in ETCC-006 and ETCC-010 DCIS cell lines compared to MCF10A, MCF10DCIS.com, MCF7 and MDA-MB-231 cell lines. RNAseq datasets of ETCC-006 and ETCC-010 were compared to datasets available in Klijn et al. (2015) [18]. Using GOseq [28], shown are plots of the top ten biological processes identified in each of the of the DCIS and breast cancer cell lines, ETCC-006, ETCC-010, MCF10DCIS.com, MCF7 and MDA-MB-231, all in comparison to MCF10A (normal-like).

**Additional file 4.**

**Additional file 5.**

**Additional file 6.**

## Data Availability

The datasets supporting the conclusions of this article are available in the European Genome-phenome Archive (EGA) repository, under study accession number EGAS00001004553, and included within the article as Additional file 1.

## Abbreviations

AKT/PKB: Protein kinase B
DCIS: Ductal carcinoma in situ
EGF: Epidermal growth factor
EGFR: Epidermal growth factor receptor
EMT: Epithelial to mesenchymal transition
ER: Estrogen receptor
ERK: Extracellular signal-regulated kinase
GAPDH: Glyceraldehyde-3-phosphate dehydrogenase
HER2: Human epidermal growth factor receptor 2
IDC: Invasive ductal carcinoma
IGF-1R: Insulin-like growth factor 1 receptor
MEK: Mitogen-activated protein kinase kinase
PDT: Population doubling time
PI3K: Phosphoinositide 3-kinase
PR: Progesterone receptor
PTEN: Phosphatase and tensin homolog
qRT-PCR: Quantitative, reverse transcriptase, polymerase chain reaction
RIN: RNA integrity
RNAseq: RNA sequencing
SEM: Standard error of mean
TNBC: Triple negative breast cancer
UBC: Polyubiquitin-C
VEGF: Vascular endothelial growth factor

## References

[1] Dai X, Cheng H, Bai Z, Li J (2017). Breast cancer cell line classification and its relevance with breast tumor subtyping. J Cancer.

[2] Holliday DL, Speirs V (2011). Choosing the right cell line for breast cancer research. Breast Cancer Res.

[3] Kao J, Salari K, Bocanegra M, Choi YL, Girard L, Gandhi J, Kwei KA, Hernandez-Boussard T, Wang P, Gazdar AF, Minna JD, Pollack JR (2009). Molecular profiling of breast Cancer cell lines defines relevant tumor models and provides a resource for Cancer gene discovery. PLoS One.

[4] Subik K, Lee J-F, Baxter L, Strzepek T, Costello D, Crowley P, Xing L, Hung MC, Bonfiglio T, Hicks DG, Tang P (2010). The expression patterns of ER, PR, HER2, CK5/6, EGFR, Ki-67 and AR by Immunohistochemical analysis in breast Cancer cell lines. Breast Cancer (Auckl).

[5] Hong YK, McMasters KM, Egger ME, Ajkay N (2018). Ductal carcinoma in situ current trends, controversies, and review of literature. Am J Surg.

[6] Cowell CF, Weigelt B, Sakr RA, Ng CKY, Hicks J, King TA, Reis-Filho JS (2013). Progression from ductal carcinoma in situ to invasive breast cancer: revisited. Mol Oncol.

[7] Gorringe KL, Fox SB. Ductal carcinoma in situ biology, biomarkers, and diagnosis. Front Oncol. 2017;7. 10.3389/fonc.2017.00248. 10.3389/fonc.2017.00248 PMC5660056 29109942

[8] Dawson PJ, Wolman SR, Tait L, Heppner GH, Miller FR (1996). MCF10AT: a model for the evolution of cancer from proliferative breast disease. Am J Pathol.

[9] Miller FR, Santner SJ, Tait L, Dawson PJ (2000). MCF10DCIS.com xenograft model of human comedo ductal carcinoma in situ. J Natl Cancer Inst.

[10] Neve RM, Chin K, Fridlyand J, Yeh J, Baehner FL, Fevr T, Clark L, Bayani N, Coppe JP, Tong F, Speed T, Spellman PT, DeVries S, Lapuk A, Wang NJ, Kuo WL, Stilwell JL, Pinkel D, Albertson DG, Waldman FM, McCormick F, Dickson RB, Johnson MD, Lippman M, Ethier S, Gazdar A, Gray JW (2006). A collection of breast cancer cell lines for the study of functionally distinct cancer subtypes. Cancer Cell.

[11] Kaur H, Mao S, Li Q, Sameni M, Krawetz SA, Sloane BF, Mattingly RR (2012). RNA-Seq of human breast ductal carcinoma in situ models reveals aldehyde dehydrogenase isoform 5A1 as a novel potential target. PLoS One.

[12] Behbod F, Kittrell FS, LaMarca H, Edwards D, Kerbawy S, Heestand JC, Young E, Mukhopadhyay P, Yeh HW, Allred DC, Hu M, Polyak K, Rosen JM, Medina D (2009). An intraductal human-in-mouse transplantation model mimics the subtypes of ductal carcinoma in situ. Breast Cancer Res.

[13] Brock EJ, Ji K, Shah S, Mattingly RR, Sloane BF (2019). In vitro models for studying invasive transitions of ductal carcinoma in situ. J Mammary Gland Biol Neoplasia.

[14] Yong JW, Choong ML, Wang S (2014). Characterization of ductal carcinoma in situ cell lines established from breast tumor of a Singapore Chinese patient. Cancer Cell Int.

[15] Soule HD, Vazguez J, Long A (1973). A human cell line from a pleural effusion derived from a breast carcinoma. J Natl Cancer Inst.

[16] Lee AV, Oesterreich S, Davidson NE (2015). MCF-7 cells--changing the course of breast Cancer research and care for 45 years. J Natl Cancer Inst.

[17] Soule HD, Maloney TM, Wolman SR, Peterson WD Jr, Brenz R, McGrath C, Russo J, Pauley RJ, Jones RF, Brooks SC (1990). Isolation and characterization of a spontaneously immortalized human breast epithelial cell line, MCF-10. Cancer Res.

[18] Klijn C, Durinck S, Stawiski EW, Haverty PM, Jiang Z, Liu H, Degenhardt J, Mayba O, Gnad F, Liu J, Pau G, Reeder J, Cao Y, Mukhyala K, Selvaraj SK, Yu M, Zynda GJ, Brauer MJ, Wu TD, Gentleman RC, Manning G, Yauch RL, Bourgon R, Stokoe D, Modrusan Z, Neve RM, de Sauvage FJ, Settleman J, Seshagiri S, Zhang Z (2015). A comprehensive transcriptional portrait of human cancer cell lines. Nat Biotechnol.

[19] Chua SL, See Too WC, Khoo BY, Few LL (2011). UBC and YWHAZ as suitable reference genes for accurate normalisation of gene expression using MCF7, HCT116 and HepG2 cell lines. Cytotechnology.

[20] Afgan E, Baker D, van den Beek M, Blankenberg D, Bouvier D, Čech M, Chilton J, Clements D, Coraor N, Eberhard C, Grüning B, Guerler A, Hillman-Jackson J, von Kuster G, Rasche E, Soranzo N, Turaga N, Taylor J, Nekrutenko A, Goecks J (2016). The galaxy platform for accessible, reproducible and collaborative biomedical analyses: 2016 update. Nucleic Acids Res.

[21] Bolger AM, Lohse M, Usadel B (2014). Trimmomatic: a flexible trimmer for Illumina sequence data. Bioinformatics.

[22] Dobin A, Davis CA, Schlesinger F, Drenkow J, Zaleski C, Jha S, Batut P, Chaisson M, Gingeras TR (2013). STAR: ultrafast universal RNA-seq aligner. Bioinformatics.

[23] Liao Y, Smyth GK, Shi W (2014). FeatureCounts: an efficient general purpose program for assigning sequence reads to genomic features. Bioinformatics.

[24] Love MI, Huber W, Anders S (2014). Moderated estimation of fold change and dispersion for RNA-seq data with DESeq2. Genome Biol.

[25] Yu G, He QY (2016). ReactomePA: an R/Bioconductor package for reactome pathway analysis and visualization. Mol BioSyst.

[26] Zaheed O, Samson J, Dean K (2020). A bioinformatics approach to identify novel long, non-coding RNAs in breast cancer cell lines from an existing RNA-sequencing dataset. Non-coding RNA Res.

[27] Anders S, Pyl PT, Huber W (2015). HTSeq--a Python framework to work with high-throughput sequencing data. Bioinformatics.

[28] Young MD, Wakefield MJ, Smyth GK, Oshlack A (2010). Gene ontology analysis for RNA-seq: accounting for selection bias. Genome Biol.

[29] Jalili V, Afgan E, Gu Q, Clements D, Blankenberg D, Goecks J, Taylor J, Nekrutenko A (2020). The galaxy platform for accessible, reproducible and collaborative biomedical analyses: 2020 update. Nucleic Acids Res.

[30] Bunnell TM, Burbach BJ, Shimizu Y, Ervasti JM (2011). β-Actin specifically controls cell growth, migration, and the G-actin pool. Mol Biol Cell.

[31] Allred DC, Wu Y, Mao S, Nagtegaal ID, Lee S, Perou CM, Mohsin SK, O'Connell P, Tsimelzon A, Medina D (2008). Ductal carcinoma in situ and the emergence of diversity during breast cancer evolution. Clin Cancer Res.

[32] Kadota M, Yang HH, Gomez B, Sato M, Clifford RJ, Meerzaman D, Dunn BK, Wakefield LM, Lee MP (2010). Delineating genetic alterations for tumor progression in the MCF10A series of breast cancer cell lines. PLoS One.

[33] Worsham MJ, Pals G, Schouten JP, Miller F, Tiwari N, van Spaendonk R, Wolman SR (2006). High-resolution mapping of molecular events associated with immortalization, transformation, and progression to breast cancer in the MCF10 model. Breast Cancer Res Treat.

[34] So JY, Lee HJ, Kramata P (2012). Differential expression of key signaling proteins in MCF10 cell lines, a human breast Cancer progression model. Mol Cell Pharmacol.

[35] van Seijen M, Lips EH, Thompson AM (2019). Ductal carcinoma in situ: to treat or not to treat, that is the question. Br J Cancer.

[36] Solin LJ, Gray R, Baehner FL, Butler SM, Hughes LL, Yoshizawa C, Cherbavaz DB, Shak S, Page DL, Sledge GW, Davidson NE, Ingle JN, Perez EA, Wood WC, Sparano JA, Badve S (2013). A multigene expression assay to predict local recurrence risk for ductal carcinoma in situ of the breast. J Natl Cancer Inst.

[37] Baldin V, Lukas J, Marcote MJ, Pagano M, Draetta G (1993). Cyclin D1 is a nuclear protein required for cell cycle progression in G1. Genes Dev.

[38] Tye BK (1999). MCM proteins in DNA replication. Annu Rev Biochem.

[39] Musa J, Aynaud M-M, Mirabeau O, Delattre O, Grünewald TGP (2017). MYBL2 (B-Myb): a central regulator of cell proliferation, cell survival and differentiation involved in tumorigenesis. Cell Death Dis.

[40] Brown LF, Guidi AJ, Schnitt SJ et al. Vascular stroma formation in carcinoma in situ, invasive carcinoma, and metastatic carcinoma of the breast. Clin Cancer Res. 1999;5(5):1041-56. 10353737

[41] Marcotte R, Sayad A, Brown KR, Sanchez-Garcia F, Reimand J, Haider M, Virtanen C, Bradner JE, Bader GD, Mills GB, Pe’er D, Moffat J, Neel BG (2016). Functional genomic landscape of human breast Cancer drivers, vulnerabilities, and resistance. Cell.

[42] Salvatorelli L, Puzzo L, Vecchio GM, et al. Ductal carcinoma in situ of the breast: an update with emphasis on radiological and morphological features as predictive prognostic factors. Cancers. 2020. 10.3390/cancers12030609. 10.3390/cancers12030609 PMC7139619 32155777

[43] Kuo DS, Labelle-Dumais C, Gould DB. COL4A1 and COL4A2 mutations and disease: insights into pathogenic mechanisms and potential therapeutic targets. Hum Mol Genet. 2012. 10.1093/hmg/dds346. 10.1093/hmg/dds346 PMC3459649 22914737

[44] Vastrad B, Vastrad C, Godavarthi A, Chandrashekar R (2017). Molecular mechanisms underlying gliomas and glioblastoma pathogenesis revealed by bioinformatics analysis of microarray data. Med Oncol.

[45] Wu Q, Zhang B, Wang Z, Hu X, Sun Y, Xu R, Chen X, Wang Q, Ju F, Ren S, Zhang C, Qi F, Ma Q, Xue Q, Zhou YL (2019). Integrated bioinformatics analysis reveals novel key biomarkers and potential candidate small molecule drugs in gastric cancer. Pathol Pract.

[46] Liu Y, Zhang J, Chen Y, Sohel H, Ke X, Chen J, Li YX (2020). The correlation and role analysis of COL4A1 and COL4A2 in hepatocarcinogenesis. Aging (Albany NY).

[47] Puck TT, Marcus PI, Cieciura SJ. Clonal growth of mammalian cells in vitro; growth characteristics of colonies from single HeLa cells with and without a feeder layer. J Exp Med. 1956;103(2):273–83. 10.1084/jem.103.2.273. 10.1084/jem.103.2.273 PMC2136583 13286432

[48] Franken NAP, Rodermond HM, Stap J, Haveman J, van Bree C (2006). Clonogenic assay of cells in vitro. Nat Protoc.

[49] Roberts AB, Roche NS, Sporn MB (1985). Selective inhibition of the anchorage-independent growth of myc-transfected fibroblasts by retinoic acid. Nature.

[50] Kalluri R, Weinberg RA (2009). The basics of epithelial-mesenchymal transition. J Clin Invest.

[51] Seyfried TN, Huysentruyt LC (2013). On the origin of cancer metastasis. Crit Rev Oncog.

[52] Derynck R, Weinberg RA (2019). EMT and Cancer: more than meets the eye. Dev Cell.

[53] Gilles C, Polette M, Mestdagt M, Nawrocki-Raby B, Ruggeri P, Birembaut P, Foidart JM (2003). Transactivation of vimentin by beta-catenin in human breast cancer cells. Cancer Res.

[54] Wang Z, Zhang H, Hou J, Niu J, Ma Z, Zhao H, Liu C (2015). Clinical implications of β-catenin protein expression in breast cancer. Int J Clin Exp Pathol.

[55] Pan B, Guo J, Liao Q, Zhao Y (2018). β1 and β3 integrins in breast, prostate and pancreatic cancer: a novel implication. Oncol Lett.

[56] Howe GA, Addison CL (2012). β1 integrin: an emerging player in the modulation of tumorigenesis and response to therapy. Cell Adhes Migr.

[57] Wee P, Wang Z. Epidermal growth factor receptor cell proliferation signaling pathways. Cancers (Basel). 2017;9(12). 10.3390/cancers9050052. 10.3390/cancers9050052 PMC5447962 28513565

[58] Denduluri SK, Idowu O, Wang Z, Liao Z, Yan Z, Mohammed MK, Ye J, Wei Q, Wang J, Zhao L, Luu HH (2015). Insulin-like growth factor (IGF) signaling in tumorigenesis and the development of cancer drug resistance. Genes Dis.

[59] Yuan TL, Wulf G, Burga L, Cantley LC (2011). Cell-to-cell variability in PI3K protein level regulates PI3K-AKT pathway activity in cell populations. Curr Biol.

[60] Vos CBJ, ter Haar NT, Peterse JL, Cornelisse CJ, van de Vijver MJ (1999). Cyclin D1 gene amplification and overexpression are present in ductal carcinomain situ of the breast. J Pathol.

[61] Lacroix M, Leclercq G (2004). Relevance of breast Cancer cell lines as models for breast Tumours: an update. Breast Cancer Res Treat.

[62] Kalaany NY, Sabatini DM (2009). Tumours with PI3K activation are resistant to dietary restriction. Nature.

[63] Barnabas N, Cohen D (2013). Phenotypic and molecular characterization of MCF10DCIS and SUM breast Cancer cell lines. Int J Breast Cancer.

[64] Vogt PK, Hart JR, Gymnopoulos M (2010). Phosphatidylinositol 3-kinase: the oncoprotein. Curr Top Microbiol Immunol.

[65] Maxwell AJ, Clements K, Hilton B, Dodwell DJ, Evans A, Kearins O, Pinder SE, Thomas J, Wallis MG, Thompson AM, Thompson A, Clements K, Dobson H, Dodwell D, Evans A, Hanby A, Hilton B, Kearins O, Lawrence G, Maxwell A, Pinder S, Sawyer E, Sibbering M, Speirs V, Thomas J, Tomlinson I, Ball G, Wallis M, Wilcox M (2018). Risk factors for the development of invasive cancer in unresected ductal carcinoma in situ. Eur J Surg Oncol.

[66] Shekhar MPV, Tait L, Pauley RJ, Wu GS, Santner SJ, Nangia-Makker P, Shekhar V, Nassar H, Visscher DW, Heppner GH, Miller FR (2008). Comedo-ductal carcinoma in situ: A paradoxical role for programmed cell death. Cancer Biol Ther.

