# Supplementary figures and images for: Molecular and cellular characterization of two patient-derived ductal carcinoma in situ (DCIS) cell lines, ETCC-006 and ETCC-010
Source: BMC Cancer. 2021 Jul 8;21:790. doi: 10.1186/s12885-021-08511-2 (PMC8268371; doi:10.1186/s12885-021-08511-2)

A

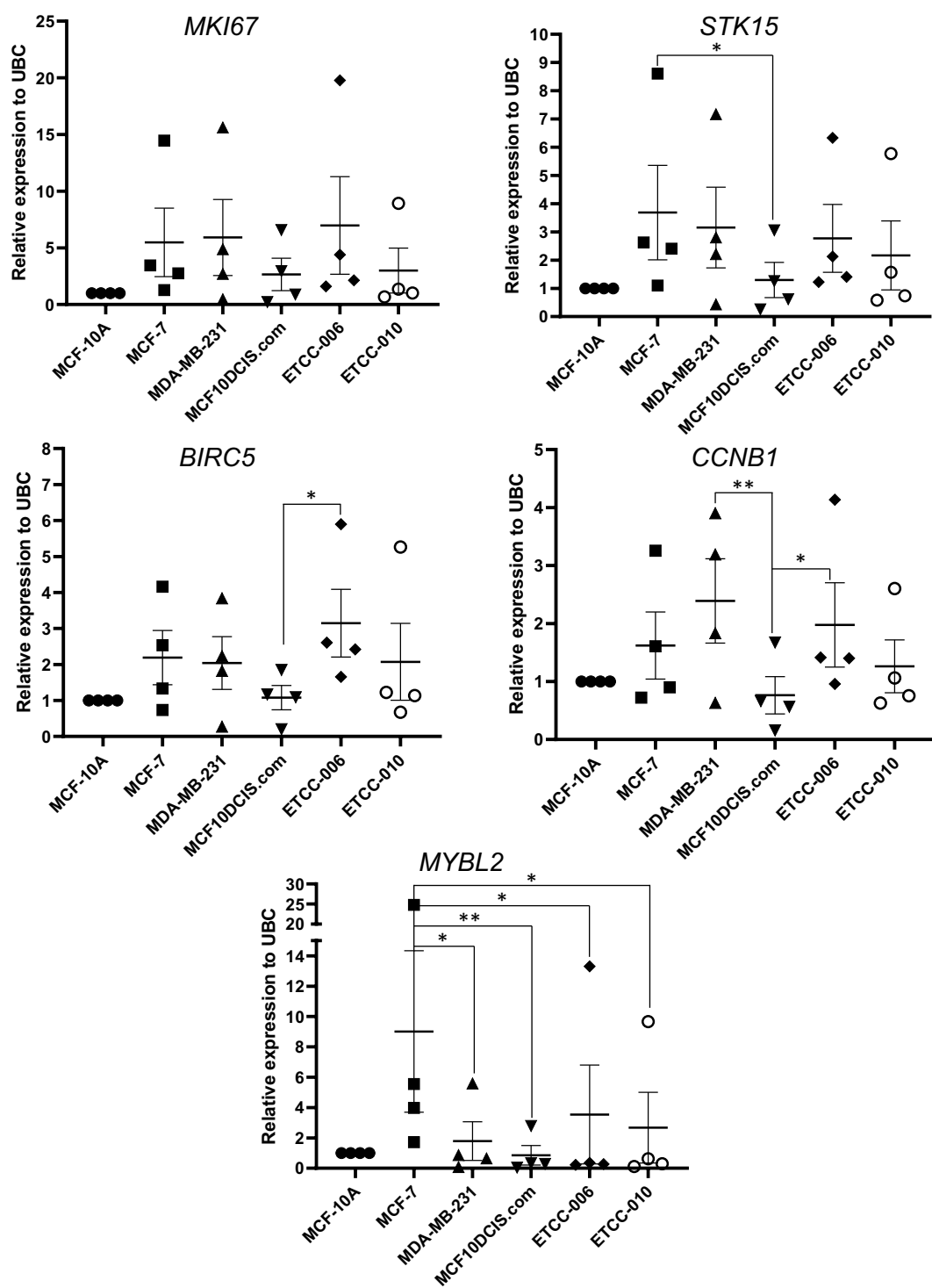

B

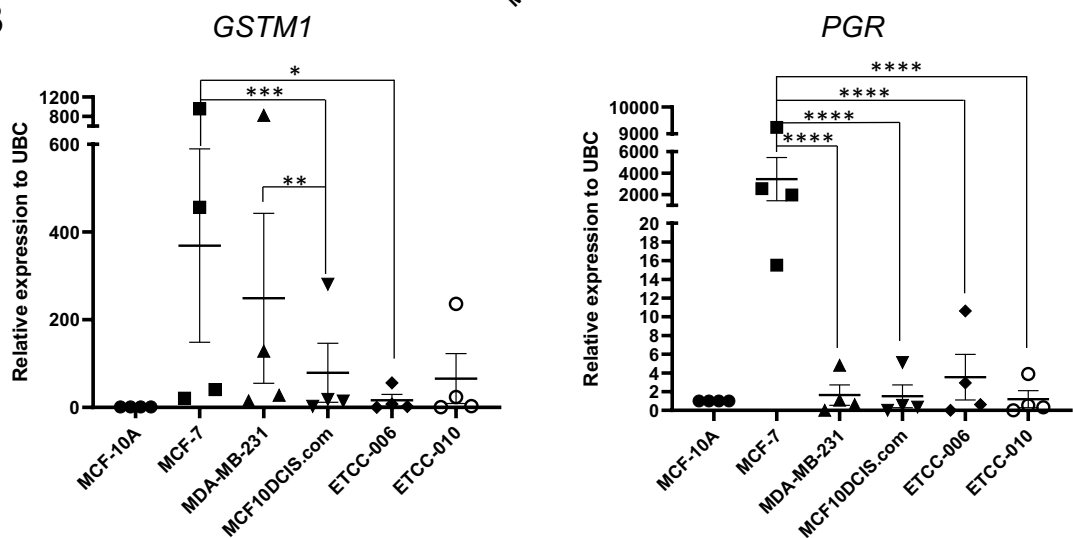

Supplement: Supplementary file 2 — Additional file 2: Supplemental Figure 1. Expression level of Oncotype DX DCIS score markers in the DCIS cell lines analyzed by qRT-PCR. (A) Relative expression of the proliferation markers is associated with an increased risk of DCIS recurrence whereas (B) GSTM1 and PGR have a protective role against the DCIS risk of recurrence. qRT-PCR was performed using cDNA synthesized from total RNA isolated from normal-like and breast cancer cell lines, MCF-10A, MCF-7, MDA-MB-231, and DCIS cell lines, MCF10DCIS.com, ETCC-006 and ETCC-010. All data was normalized to UBC (polyubiquitin-C) and statistical analysis was done using one-way ANOVA, with **** p-value < 0.0001; *** p-value < 0.001; ** p-value < 0.01; * p-value < 0.05. Horizontal bars show the mean +/− SEM (GraphPad Prism v.8.3.0). [file 12885_2021_8511_MOESM2_ESM.pdf]

# ETCC-006

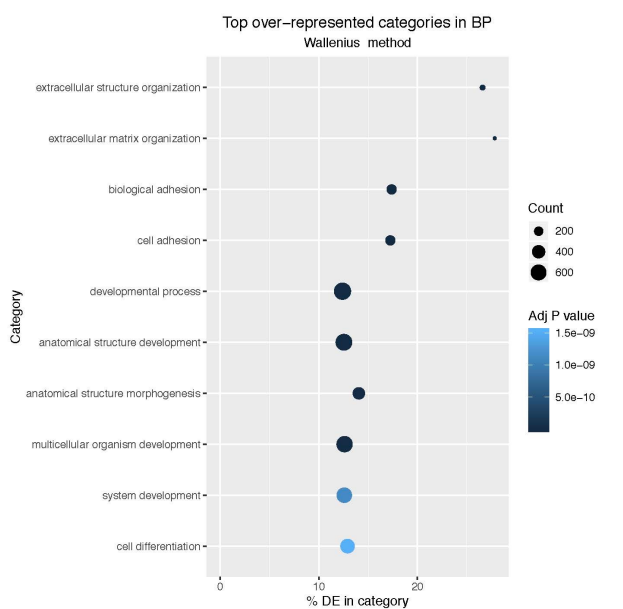

# ETCC-010

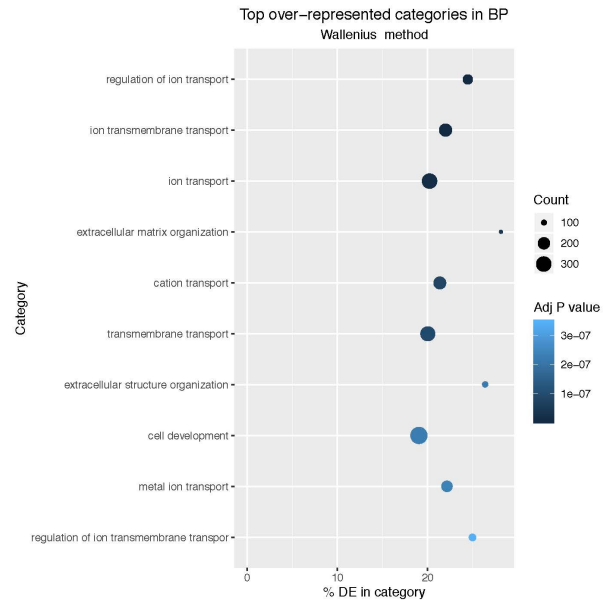

# MCF10DCIS.com

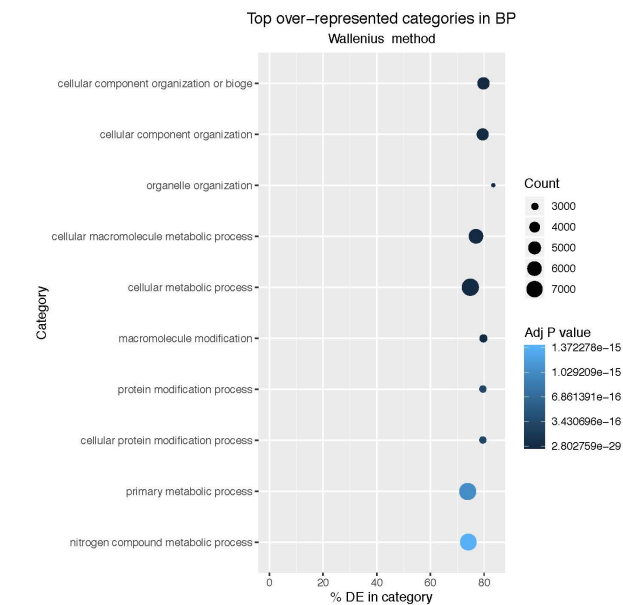

# MCF7

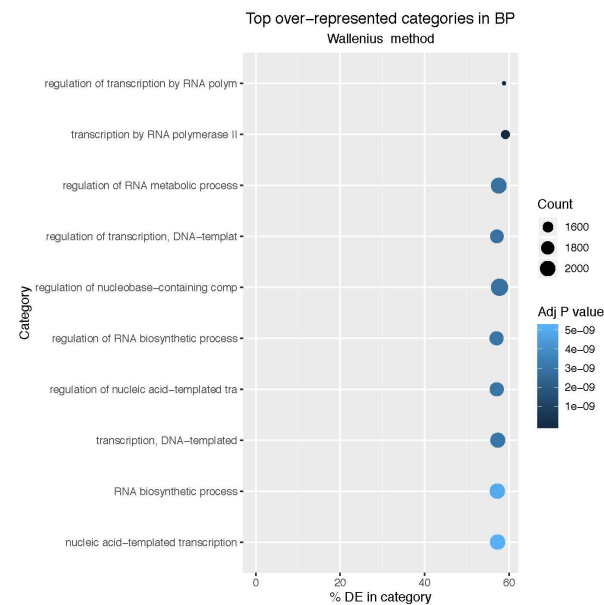

# MDA-MB-231

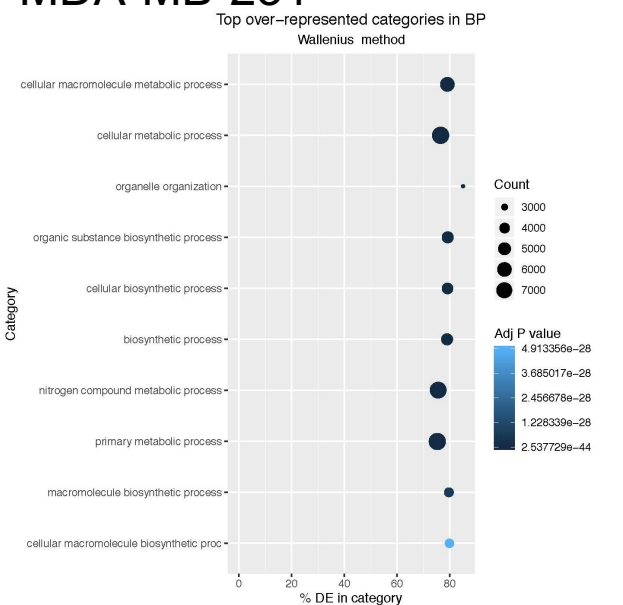

Supplement: Supplementary file 3 — Additional file 3: Supplemental Figure 2. GO analysis from the differential expression of RNA transcripts in ETCC-006 and ETCC-010 DCIS cell lines compared to MCF10A, MCF10DCIS.com, MCF7 and MDA-MB-231 cell lines. RNAseq datasets of ETCC-006 and ETCC-010 were compared to datasets available in Klijn et al. (2015) [18]. Using GOseq [28], shown are plots of the top ten biological processes identified in each of the of the DCIS and breast cancer cell lines, ETCC-006, ETCC-010, MCF10DCIS.com, MCF7 and MDA-MB-231, all in comparison to MCF10A (normal-like). [file 12885_2021_8511_MOESM3_ESM.pdf]

Figure 3: original blots of ER $\alpha$ , PR, HER2 and  $\beta$  actin

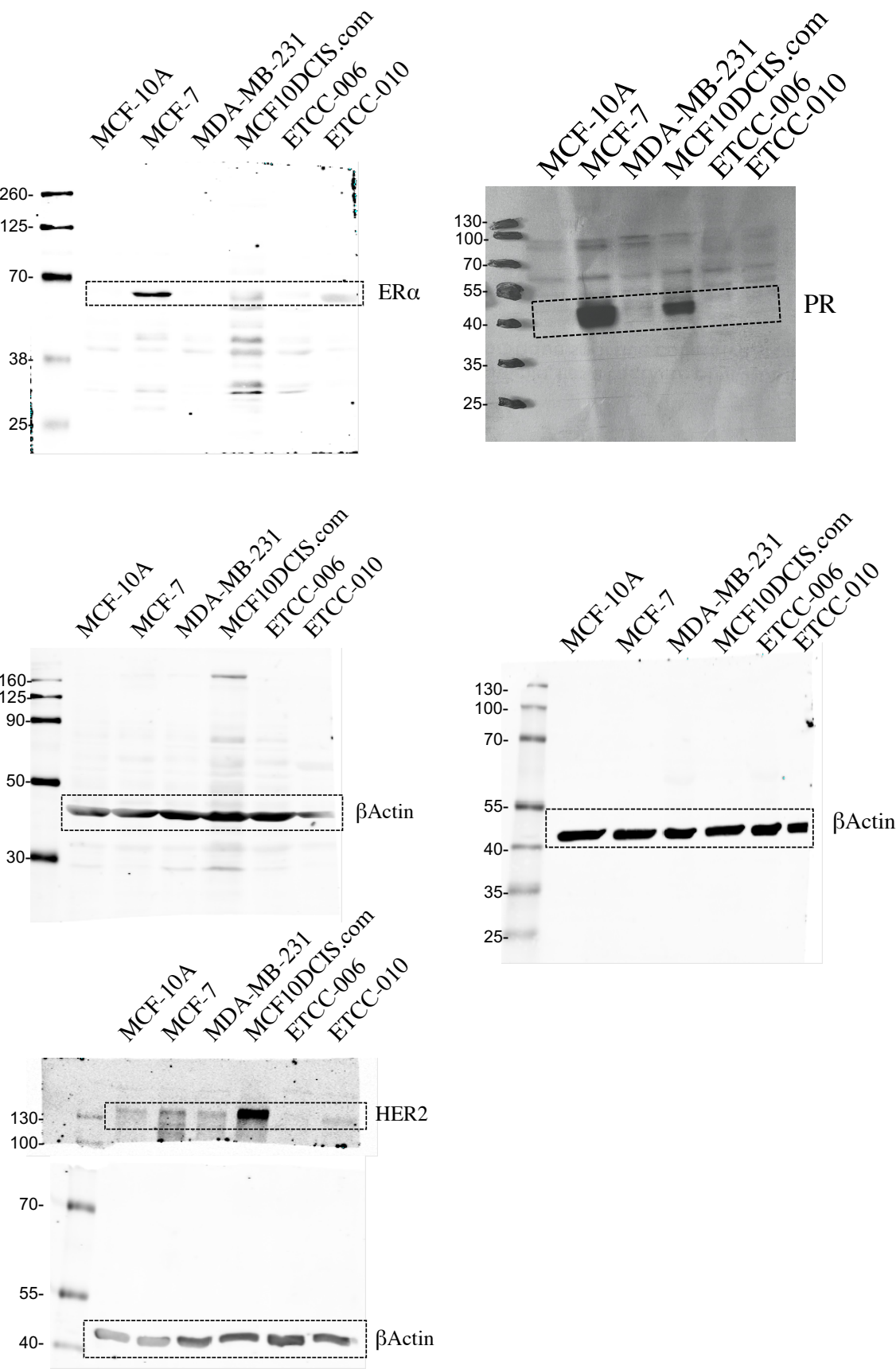

Supplement: Supplementary file 4 — Additional file 4. [file 12885_2021_8511_MOESM4_ESM.pdf]

Figure 7: original blots for  $\beta$  catenin, vimentin and  $\beta$  actin

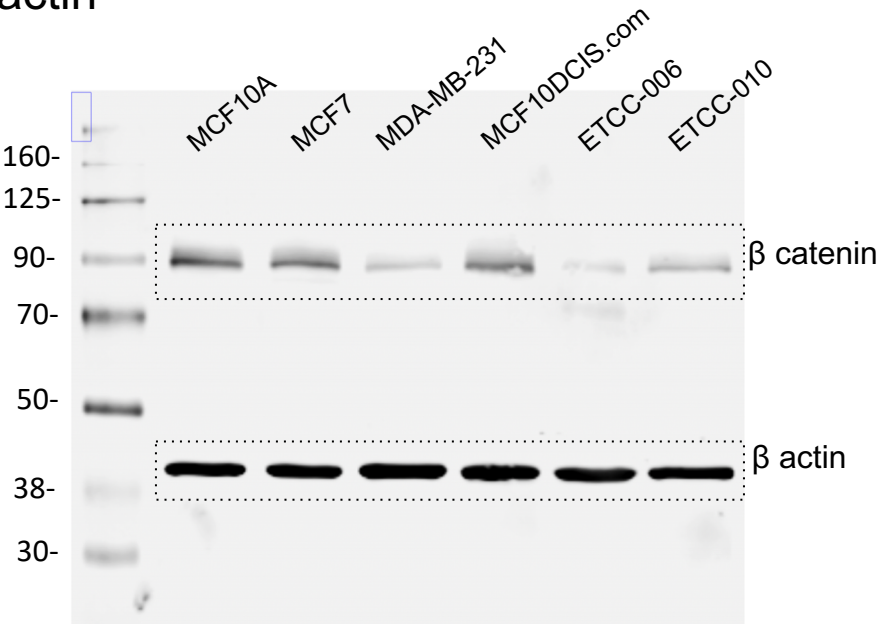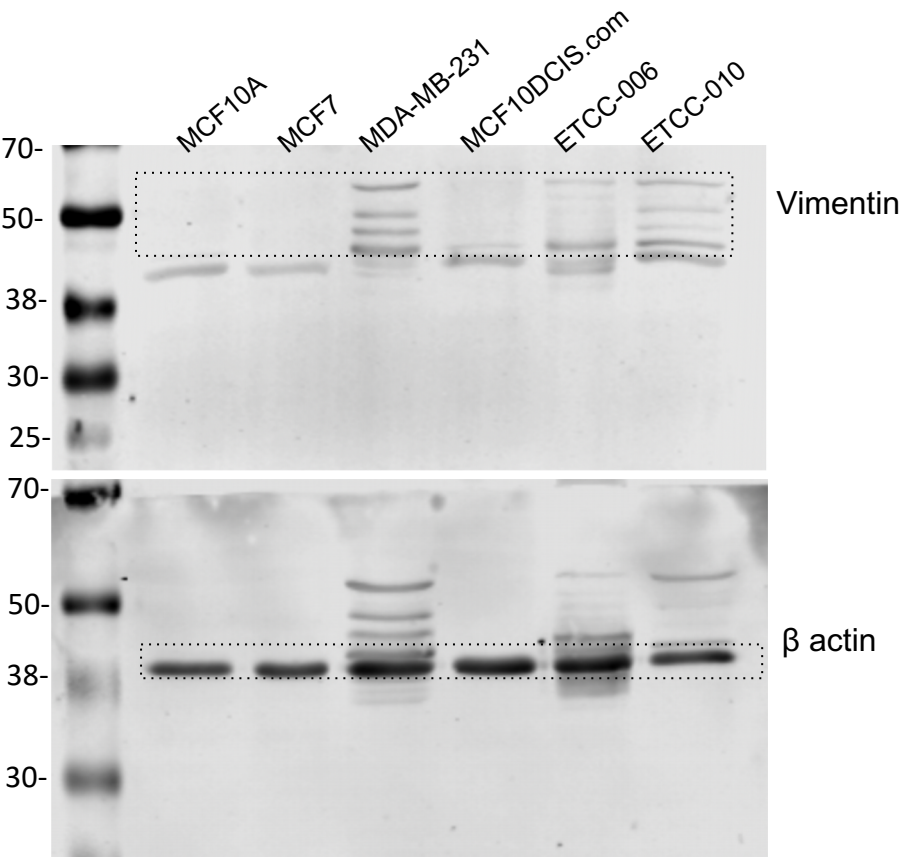

Supplement: Supplementary file 5 — Additional file 5. [file 12885_2021_8511_MOESM5_ESM.zip › Supp Fig 7/SuppMat_Fig7REVa_June2021R2.pdf]

Figure 7: original blots integrin  $\beta 1$ , E-cadherin, N-cadherin and GAPDH

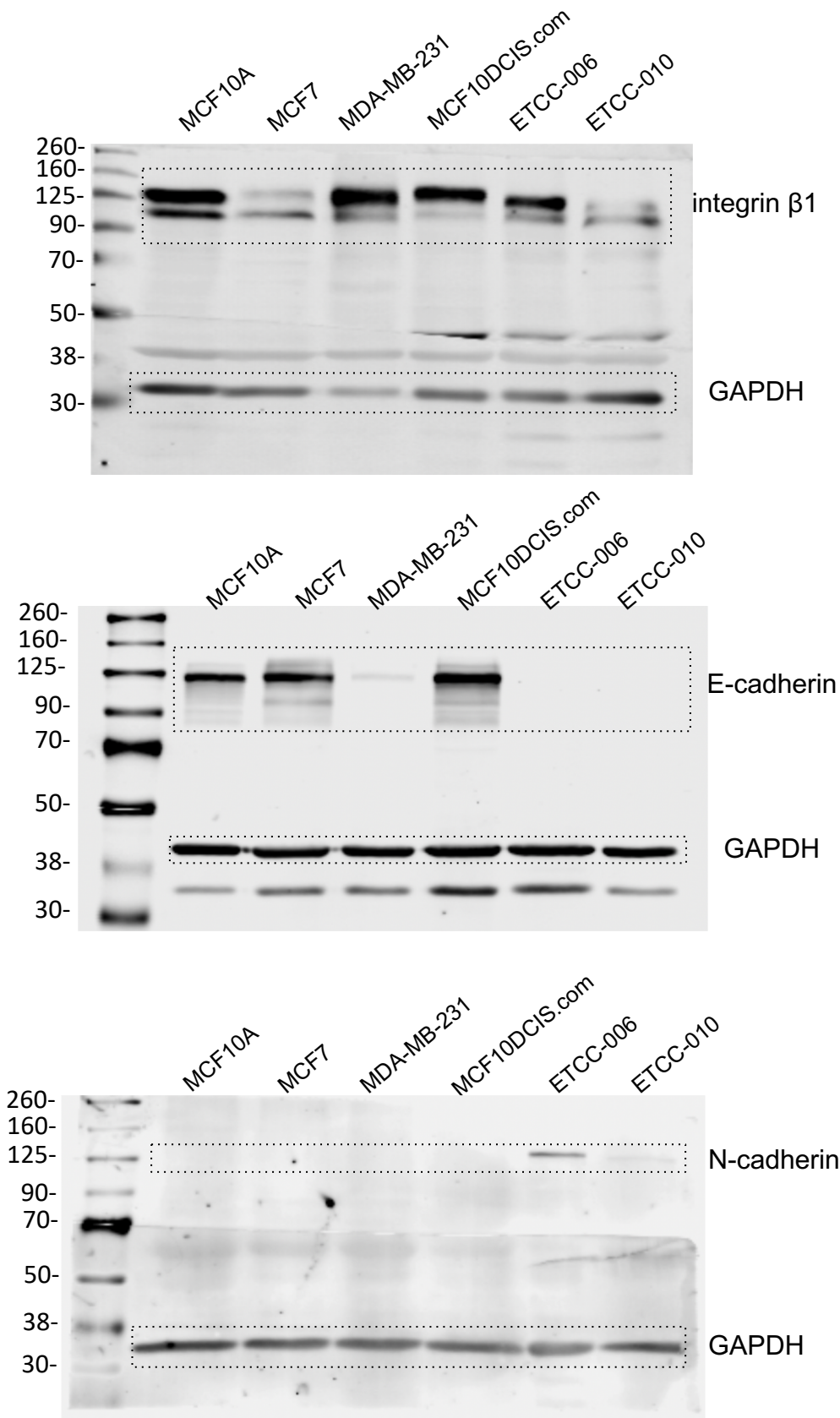

Supplement: Supplementary file 5 — Additional file 5. [file 12885_2021_8511_MOESM5_ESM.zip › Supp Fig 7/SuppMat_Fig7REVb_June2021R2.pdf]

Figure 8A: original blots for EGFR, pEGFR, IGFR and  $\beta$  actin

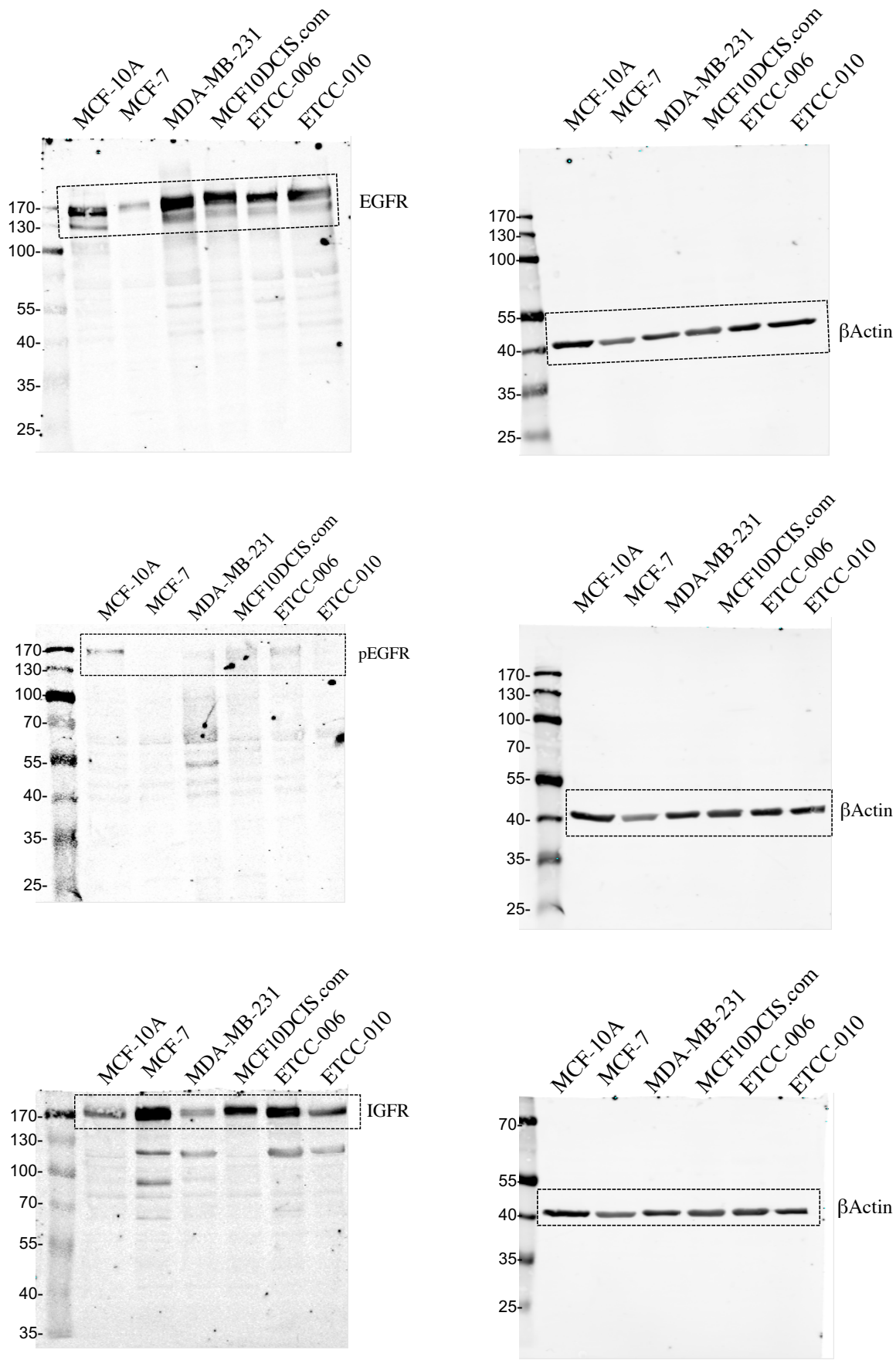

Supplement: Supplementary file 6 — Additional file 6. [file 12885_2021_8511_MOESM6_ESM.zip › Supp Fig 8/SuppMat_Fig8A_June2021R2.pdf]

Figure 8C: original blots for AKT, pAKT, GAPDH, ERK, pERK and  $\beta$  actin

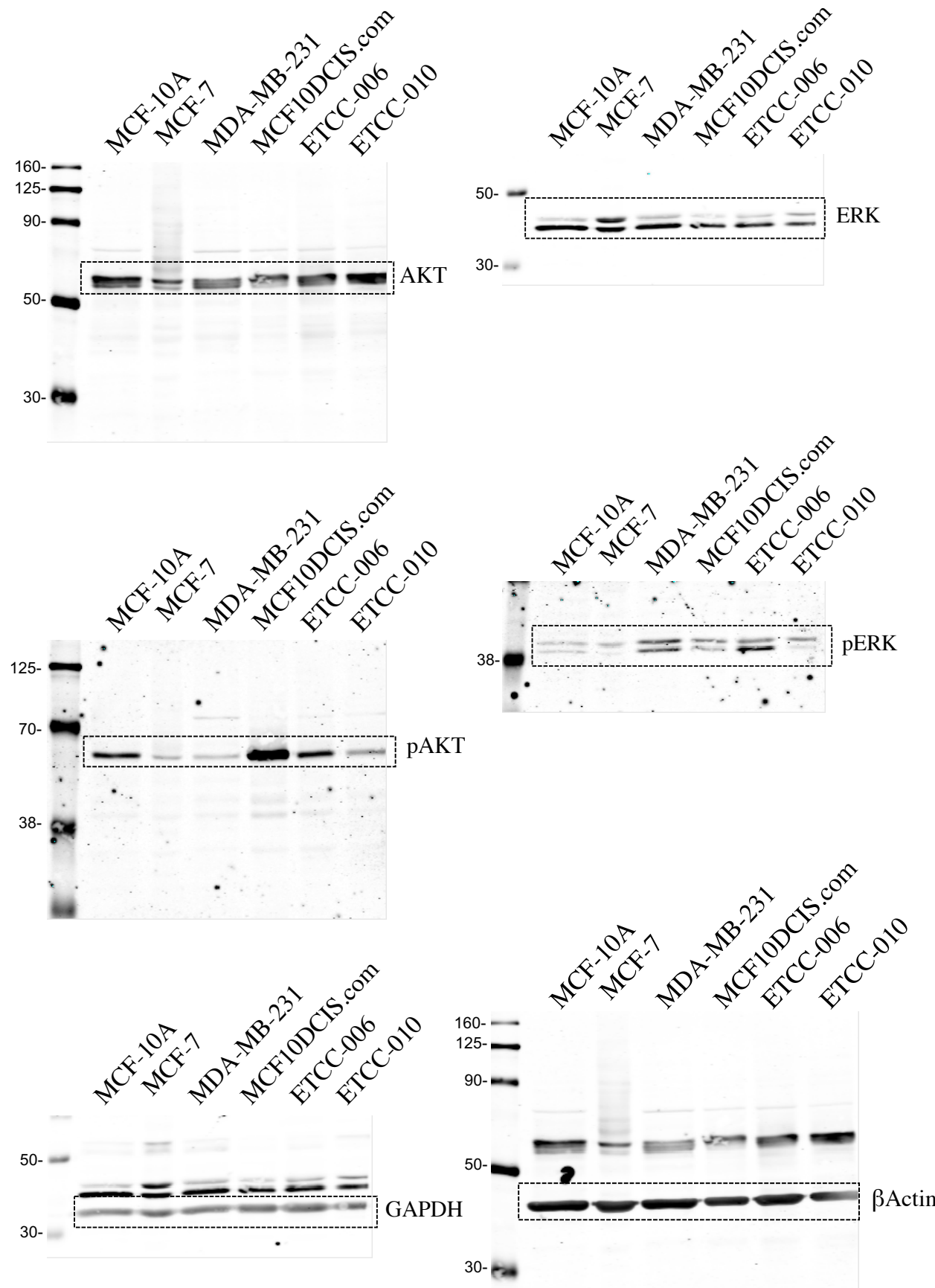

Supplement: Supplementary file 6 — Additional file 6. [file 12885_2021_8511_MOESM6_ESM.zip › Supp Fig 8/SuppMat_Fig8C_June2021R2.pdf]

Figure 8E: original blots for AKT, pAKT, ERK, pERK and GAPDH

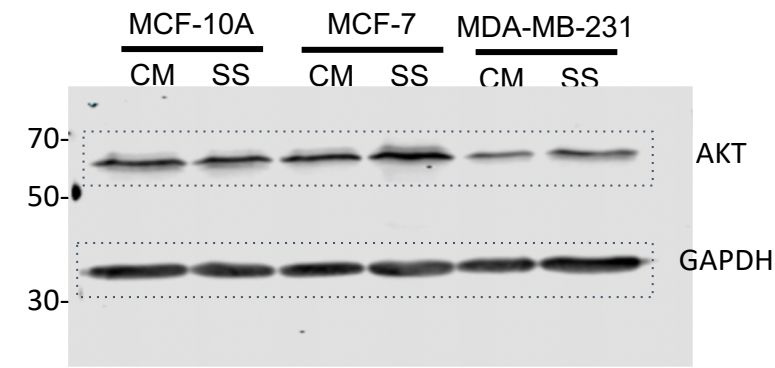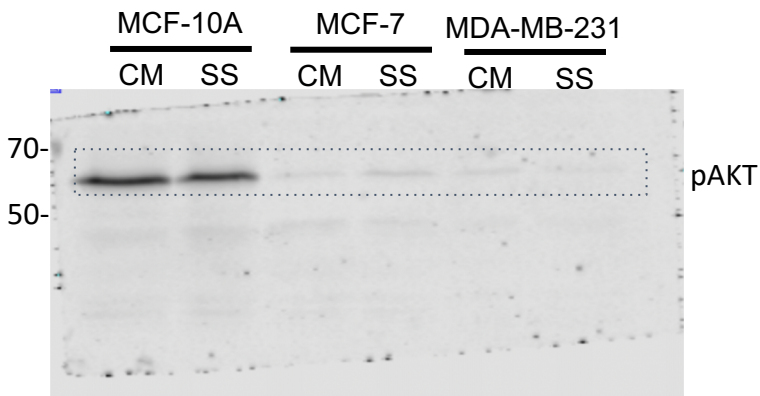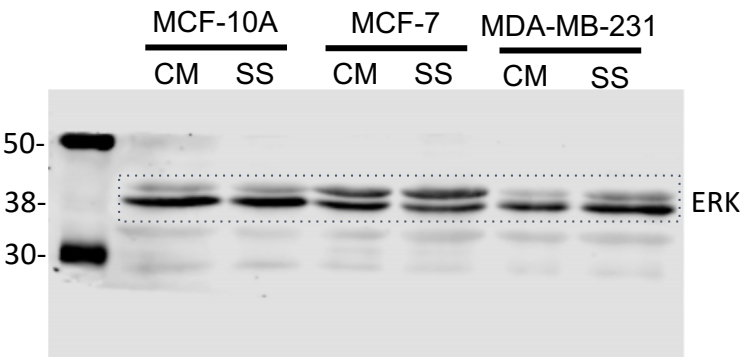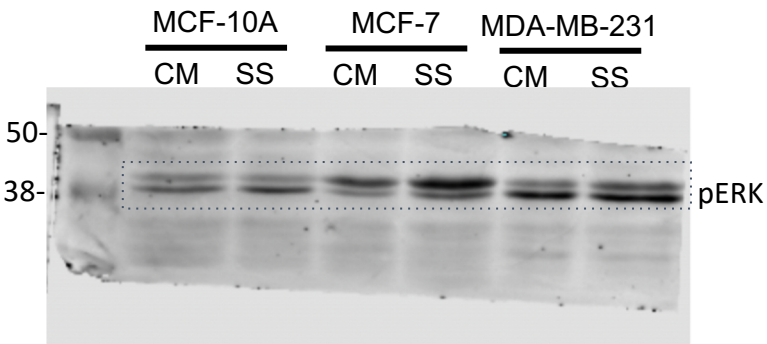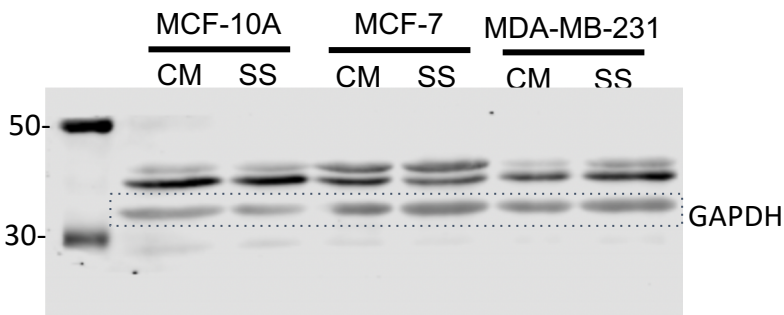

Supplement: Supplementary file 6 — Additional file 6. [file 12885_2021_8511_MOESM6_ESM.zip › Supp Fig 8/SuppMat_Fig8EREV_June2021R2.pdf]

Figure 8F: original blots for AKT, pAKT, ERK, pERK and GAPDH

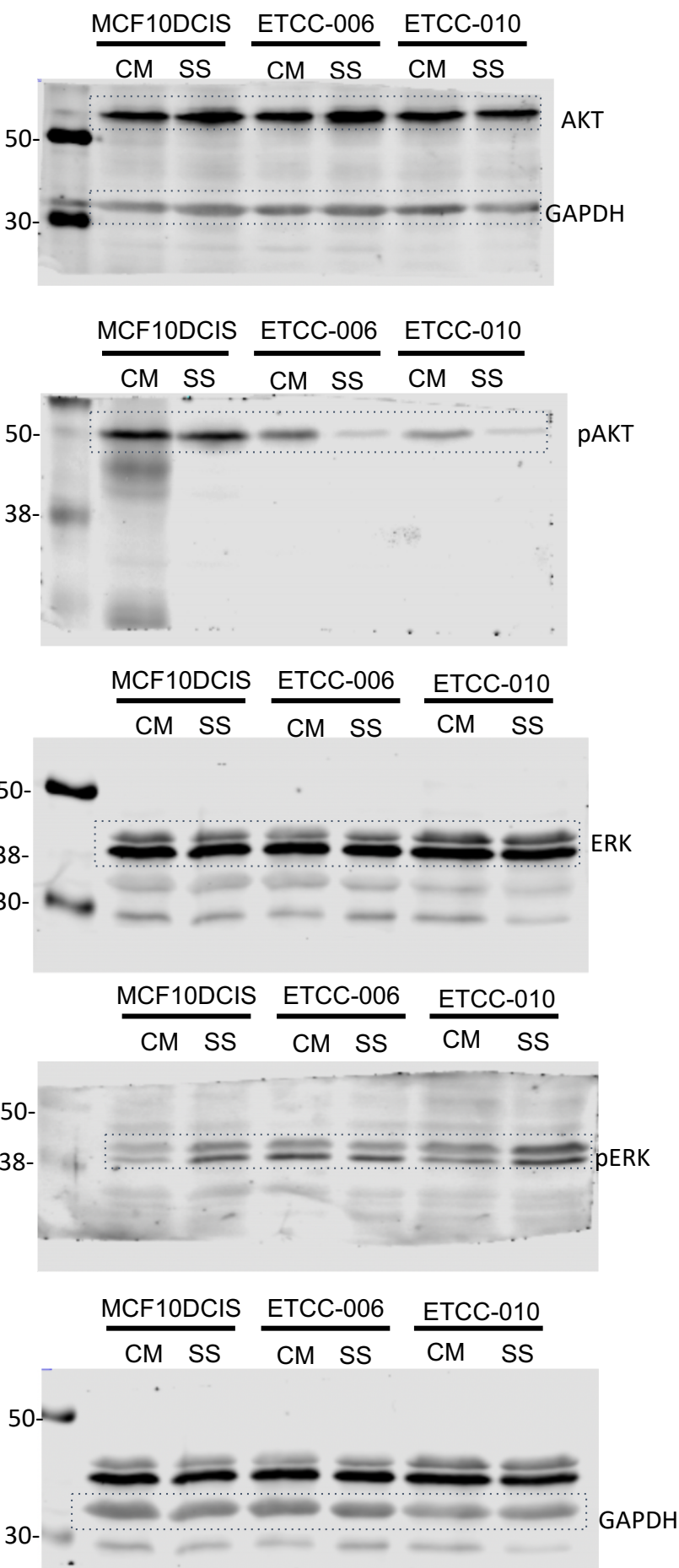

Supplement: Supplementary file 6 — Additional file 6. [file 12885_2021_8511_MOESM6_ESM.zip › Supp Fig 8/SuppMat_Fig8FREV_June2021R2.pdf]

Figure 8H: original blots for cyclin B1, GAPDH, cyclin D1 and  $\beta$  actin

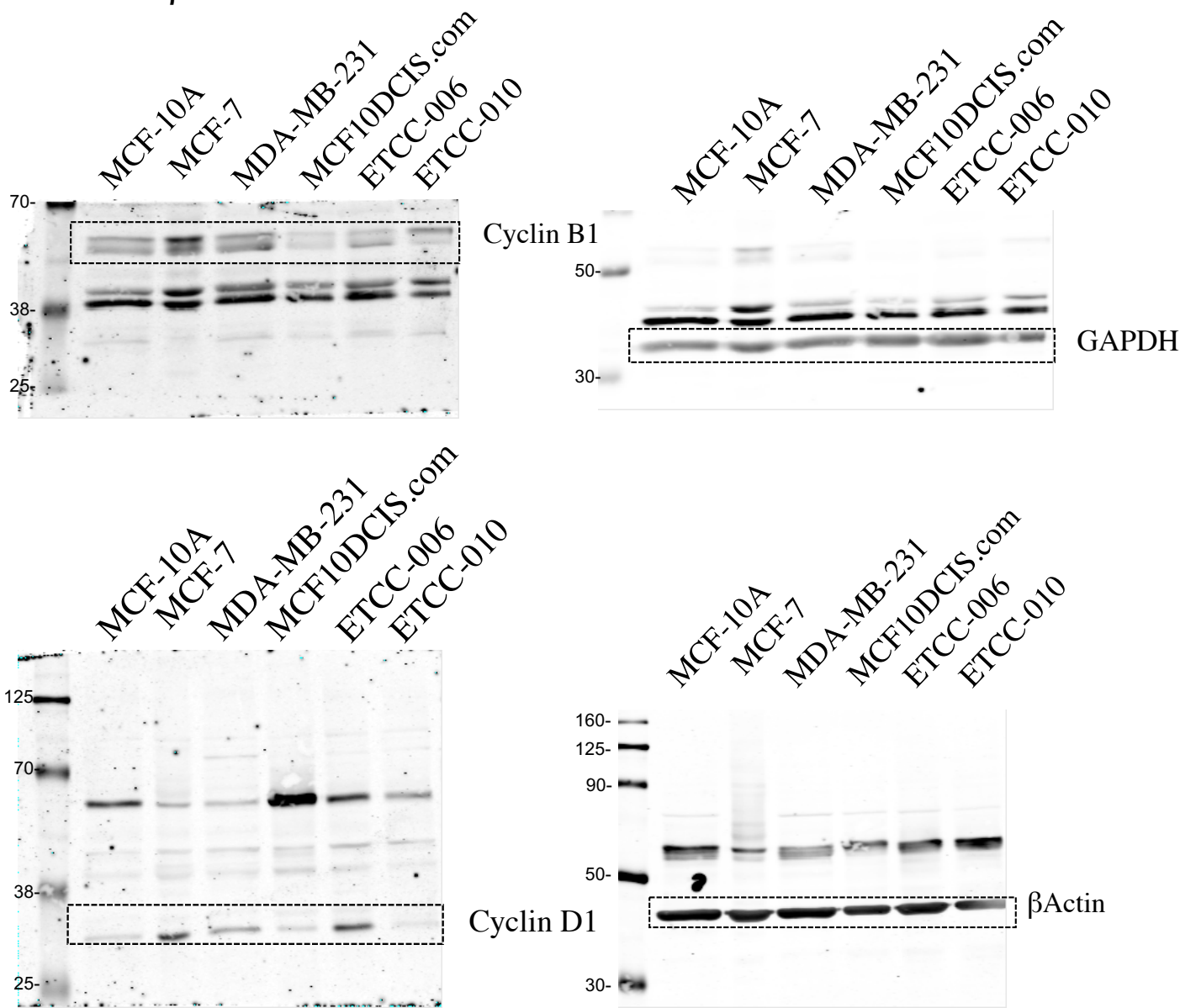

Supplement: Supplementary file 6 — Additional file 6. [file 12885_2021_8511_MOESM6_ESM.zip › Supp Fig 8/SuppMat_Fig8H_June2021R2.pdf]
